# Left-sided heart failure burden and mortality in idiopathic pulmonary fibrosis: a population-based study

**DOI:** 10.1186/s12890-022-01973-5

**Published:** 2022-05-12

**Authors:** Ardita Koteci, Ann D. Morgan, Laura Portas, Hannah R. Whittaker, Constantinos Kallis, Peter M. George, Jennifer K. Quint

**Affiliations:** 1 National Heart and Lung Institute, Imperial College London, Emmanuel Kaye Building, 1B Manresa Road, London, SW3 6LR UK; 2 NIHR Imperial Biomedical Research Centre, London, UK; 3 Royal Brompton Hospital, London, UK

**Keywords:** Idiopathic pulmonary fibrosis, Left-sided heart failure, Epidemiology

## Abstract

**Background:**

Cardiovascular disease is prevalent in idiopathic pulmonary fibrosis (IPF), yet the extent of left-sided heart failure (HF) burden, whether this has changed with time and whether HF impacts mortality risk in these patients are unknown. The aims of this study were therefore to determine the temporal trends in incidence and prevalence of left-sided HF in patients with IPF in England and compare these to published estimates in the general population and those with comparable chronic respiratory conditions such as chronic obstructive pulmonary disease (COPD), as well as determine the risk of all-cause and cause-specific mortality in patients with comorbid left-sided HF and IPF at population-level using electronic healthcare data.

**Methods:**

Clinical Practice Research Datalink (CPRD) Aurum primary-care data linked to mortality and secondary-care data was used to identify IPF patients in England. Left-sided HF prevalence and incidence rates were calculated for each calendar year between 2010 and 2019, stratified by age and sex. Risk of all-cause, cardiovascular and IPF-specific mortality was calculated using multivariate Cox regression.

**Results:**

From 40,577patients with an IPF code in CPRD Aurum, 25, 341 IPF patients met inclusion criteria. Left-sided HF prevalence decreased from 33.4% (95% CI 32.2–34.6) in 2010 to 20.9% (20.0–21.7) in 2019. Left-sided HF incidence rate per 100 person-years (95% CI) remained stable between 2010 and 2017 but decreased from 4.3 (3.9–4.8) in 2017 to 3.4 (3.0–3.9) in 2019. Throughout follow-up, prevalence and incidence were higher in men and with increasing age. Comorbid HF was associated with poorer survival (adjusted HR (95%CI) 1.08 (1.03–1.14) for all-cause mortality; 1.32 (1.09–1.59) for cardiovascular mortality).

**Conclusion:**

Left-sided HF burden in IPF patients in England remains high, with incidence almost 4 times higher than in COPD, a comparable lung disease with similar cardiovascular risk factors. Comorbid left-sided HF is also a poor prognostic marker. More substantial reduction in left-sided HF prevalence than incidence suggests persistently high IPF mortality. Given rising IPF incidence in the UK, this calls for better management of comorbidities such as left-sided HF to help optimise IPF survival.

**Supplementary Information:**

The online version contains supplementary material available at 10.1186/s12890-022-01973-5.

## Background

Idiopathic pulmonary fibrosis (IPF) is a chronic, progressive disease resulting in irreversible lung scarring. Due to diagnostic challenges, evolving diagnostic criteria as well as differences in study methodologies, estimates of IPF disease burden show substantial heterogeneity, ranging from a prevalence of 0.57–4.51 per 10,000 persons in Asia, 0.33–2.51 in Europe, and 2.40–2.98 in North America [1]. Although IPF incidence estimates also vary between studies and geographical regions, a consistent finding is that its incidence is rising both in the UK and globally [2]. With IPF onset often in older age, coexistent multimorbidity is common, with over 80% of IPF patients having at least one or more co-morbidity [3–5] A consistent finding amongst observational studies is that cardiovascular disease (CVD) is the most prevalent co-morbidity and furthermore a significant risk factor for increased mortality [5, 6]. For instance, an analysis of the British Thoracic Society (BTS) IPF registry between 2013 and 2019 highlighted that 34% of patients had hypertension and 21% had ischaemic heart disease (IHD) [7]. Similar findings have been echoed in cohorts from several other countries, with 77.9% of patients having concurrent CVD in a German tertiary centre [5] and 63% in a Danish cohort [6]. While CVD can exist prior to IPF onset, IPF itself has also been shown to be an independent risk factor for CVD, even after adjusting for common cardiovascular risk factors [8–10] Furthermore, incident CVD risk is comparatively higher in IPF than other chronic respiratory diseases such as chronic obstructive pulmonary disease (COPD) [11] and gender differences have been noted, with a higher risk of comorbid CVD in men than women [12].

However, while the high burden of CVDs such as IHD [8], pulmonary hypertension and right heart failure (HF) [13] in IPF are well-documented, evidence of left-sided HF burden and its association with mortality is, by contrast, sparser. Given that CVDs such as IHD, hypertension and atrial fibrillation can all contribute to the pathophysiology of left-sided HF and that both IPF and HF share similar pathophysiological mechanisms such as cellular senescence [14, 15], it unsurprising that IPF and HF often co-exist. Indeed echocardiographic studies of IPF patients have shown left ventricular (LV) diastolic dysfunction [16], and presence of HF with either preserved (HFpEF) or reduced (HFrEF) ejection fraction [17]. Furthermore, observational studies in the US have noted a high prevalence of HF in patients with IPF, ranging between 11 and 20% depending on the population studied [18, 19]. This is notably up to twelve times higher than in the general population in the UK [20, 21] and four times higher than estimates in patients with other chronic respiratory conditions such as COPD [22]. Similarly, left-sided HF has been shown to be a poor prognostic marker which negatively impacts survival of IPF patients [5, 12, 23]. Given the mean overall survival of IPF patients remains 4 years in the absence of antifibrotic treatment [24] and in the face of increasing IPF mortality in the UK [25], the increased mortality risk which comorbid HF adds in IPF is therefore significant given the high burden of HF in these patients.

While there is evidence that IPF and left-sided HF co-exist, there are few population-level studies examining the epidemiology of left-sided HF in IPF patients, with generalizability of findings from existing studies made challenging by use of small cohorts from registries or single centres, frequent study of HF in only hospitalised patients, and lack of clear HF case definition [26]. Furthermore, temporal changes in HF incidence and prevalence have been shown in the general population in England, with a moderate decline in its standardised incidence yet increasing absolute numbers of both incident and prevalent heart failure cases [21]. By contrast, no studies have explored whether similar temporal trends exist within the context of IPF. Given the rising incidence of IPF both in the UK and globally as well as the rising absolute burden of HF in the UK population over time, understanding the scale of the problem in IPF is important to guide appropriate allocation of healthcare resources to address the management of co-morbidities, with this having been shown to be key to optimising IPF survival [13, 27]. Furthermore, the treatment landscape of IPF has changed considerably in the last decade, including the introduction of antifibrotic therapies such as pirfenidone and nintedanib in 2013 and 2016 in the UK, respectively, which have been shown to have some cardioprotective properties outside the context of IPF [28, 29] and the discontinuation of long-term use of medications with a cardiovascular risk profile such as steroids following deleterious outcomes in the PANTHER-IPF trial in 2012 [30]. In the absence of any temporal data on the burden of left -sided HF in IPF patients, it is unknown whether this has changed over time in response to changing treatment patterns.

The aim of this study was therefore to investigate temporal trends in incidence and prevalence of left-sided HF in IPF patients in England, compare this to published estimates in the general population and those with comparable chronic respiratory conditions such as chronic obstructive pulmonary disease (COPD), as well as explore the risk of all-cause, cardiovascular and IPF-specific mortality in England using population-level electronic healthcare data. This is the first European study to explore temporal patterns of the burden of left-sided HF in IPF and one of few studies to date to investigate left-sided HF epidemiology in IPF using nationally representative population-level data.

## Method

### Data source

Data were obtained from the Clinical Practice Research Datalink (CPRD) Aurum, a nationally- representative database of de-identified primary care electronic healthcare records (EHR) covering approximately 19% of the UK population [31]. Use of CPRD Aurum for population health research has been extensively validated [32]. Two systematic reviews identified over 250 validation studies of CPRD data [33, 34] and noted high validity of most diagnoses. For instance, a review of 357 validations investigating 183 different diagnoses noted a median 88% of respiratory diseases and 85.3% of circulatory system diseases were validated [33]. Similarly, high positive predictive values (PPV) for several chronic disease diagnoses have been noted in CPRD [34]. This ranges from a PPV of 92.7% for cerebrovascular disease, 64.4% for atrial fibrillation and 98.6% for diabetes [34]. Beyond diagnoses, prescription data in CPRD is known to be well documented as GPs use the software to generate prescriptions which are automatically recorded in the database [33]. Beyond the external validity of diagnoses, CPRD also undertakes various levels of internal data validation and quality assurance, with validation occurring at three different levels: the collection stage to ensure data received from general practices is of the correct type and format, the transformation stage to ensure each record links to a patient, and at a research-quality level [32]. CPRD Aurum subsequently generates an ‘acceptability’ flag to highlight which records are of sufficient quality for research purposes and in this study, we have only included patients in the cohort if they were deemed to have an acceptable record for research purposes. The internal validation process described above consists of over 900 checks of the integrity, structure and format of the data, with any issues identified being addressed prior to inclusion of data into CPRD Aurum [32].

CPRD Aurum data for eligible patients registered in England was linked to secondary care data from Hospital Episode Statistics (HES), mortality data from Office of National Statistics (ONS) and socioeconomic data from the 2015 Index of Multiple Deprivation (IMD). Data linkage between sources was conducted by NHS Digital, a statutory trusted third party.

### Study population

Consistent with previous studies exploring IPF epidemiology by means of diagnostic codes in EHR [35, 36], we adopted the term IPF-clinical syndrome (IPF-CS) rather than IPF alone due to heterogeneity of IPF coding terms. Adults with IPF-CS diagnosed after 40 years of age, with at least 12 months of primary care data prior to cohort entry and eligible for linkage with HES, ONS and IMD, with a record of acceptable quality for research purposes were eligible for inclusion. IPF-CS diagnosis was identified by use of a codelist screened by clinicians (PMG, JKQ), with inclusion of terms guided by definitions used in previous literature [35, 36], and IPF diagnostic guidelines [37]. The full codelist is accessible at: https://github.com/NHLI-Respiratory-Epi/IPF_HF_codelists.

### Study design

We used a retrospective cohort study design with rolling entry and a 10-year study period (1^st^ January 2010–31st December 2019). For each patient, start of follow-up was defined as the latest of: 12 months post registration date with a general practice, start of study, patient’s 40^th^ birthday or date of IPF-CS diagnosis. The end of follow-up was defined as the earliest of: transfer out of practice, end of study, last data collection date or patient’s date of death.

### Covariates

Ethnicity data was obtained from linked HES data, while socioeconomic status was obtained from linked IMD records. Codelists screened by a clinician (JKQ) and based on previously published work were used to identify baseline covariates. Smoking, alcohol status and body mass index (BMI) were determined closest to start of follow-up. BMI data in the 3 years prior to start of follow-up was used, with BMI values calculated from height and weight data closest to start of follow-up, where available, in event of missing BMI data. Presence of baseline cardiovascular comorbidities and left-sided HF risk factors were determined at any point prior to start of follow-up, while cardiovascular medication use was defined as at least one prescription in the 12 months preceding start of follow-up. All codelists used, including those for ascertaining outcomes below, are accessible at: https://github.com/NHLI-Respiratory-Epi/IPF_HF_codelists

### Outcome ascertainment

Codelists were also used to identify prevalent and incident left-sided HF. Codes denoting right HF were excluded.

Cause of death was ascertained from ICD-10 codes using the derived cause of death field in linked ONS records. All-cause mortality was defined as death from any cause, with cardiovascular mortality defined as death due to myocardial infarction, ischaemic stroke or HF.

### Statistical analysis

Baseline characteristics are presented as absolute numbers and frequencies (%) for categorical data and medians with interquartile ranges [IQR] for non-normally distributed continuous variables. Proportions of missing data are described as frequencies (%) for each covariate.

Left-sided HF prevalence and crude incidence rates were calculated annually for the 10-year study period, stratified by age (40–59, 60–79 and >  = 80) and sex. Incidence rates are reported per 100 person-years at risk, with associated 95% confidence intervals (CI).

Multivariate Cox regression was used for mortality analyses. Covariate inclusion in the multivariate models was based on directed acyclic graphs (DAGs) created using clinical knowledge and existing literature to identify possible confounders (Additional file 1: Fig. S1-3). All identified confounders were included in the models to allow development of multivariate models with most clinical utility. Patients with missing data for included covariates were excluded from analyses. The proportional hazards assumption was tested graphically for each model, with further testing using Schoenfeld residuals where necessary. Crude and adjusted hazard ratios (HR) with associated 95% CI are presented for each analysis. A *p* < 0.05 level of statistical significance was used in all analyses. All analyses were conducted in Stata v16.0 (StataCorp. 2019. *Stata Statistical Software: Release 16*. College Station, TX: StataCorp LLC).

### Sensitivity analyses

To determine whether incidence and prevalence estimates were affected by IPF-CS case definitions, we conducted the following sensitivity analyses: (1) removing patients from the cohort diagnosed with IPF-CS post 1st September 2018 when ATS/ERS/JRS/ALAT IPF diagnostic guidelines changed (2) removing patients with non-specific IPF-CS diagnostic codes such as fibrosis of lung, pulmonary fibrosis, diffuse pulmonary fibrosis, Hamman-Rich syndrome, O/E-fibrosis of lung; (3) removing patients if they had evidence of another cause for pulmonary fibrosis or interstitial lung disease other than IPF, based on ICD-10 codes denoting an autoimmune condition, occupational lung disease, hypersensitivity pneumonitis or sarcoidosis in mortality data; and (4) removing patients diagnosed with IPF-CS when younger than 50 or 60 years old to exclude possible hereditary or familial IPF cases.

For mortality analyses, ethnicity and IMD were also added as possible confounders in the multivariate models for all-cause and cardiovascular mortality.

## Results

25,341 IPF-CS patients from 1379 general practices in England were included in the cohort (Additional file 1: Table S4). Median age was 76.4 years (IQR 68.7–82.5) and 60.3% were male. 79.8% had a non-specific code for pulmonary fibrosis (Additional file 1: Table S5). Mean duration of IPF-CS prior to start of follow-up was 1.6 years (SD ± 3.48) and median age at diagnosis was 74.9 years (IQR 66.8–81.4). Median follow-up was 2.2 years.

5032 patients had a left-sided HF diagnosis, of which 57.4% (2888) had HF diagnosed prior to IPF-CS, 3.7% were diagnosed concurrently with IPF-CS and 38.9% following IPF-CS.

Those with comorbid IPF-CS and left-sided HF were more likely to be older, male, more socioeconomically deprived, have a higher BMI, be an ex-smoker, have a history of CVD or HF risk factors and be on cardiovascular medications at start of follow-up (Table 1).

**Table 1:** Baseline cohort characteristics of IPF-CS patients with and without left-sided HF

	IPF-CS -no HF (N = 20,309) n	(%)	IPF-CS + HF (N = 5032) n	(%)
*Age (years)*				
40–49	404	(2.0)	35	(0.7)
50–59	1541	(7.6)	145	(2.9)
60–69	4367	(21.5)	672	(13.4)
70–79	7536	(37.1)	1869	(37.1)
80–89	5664	(27.9)	1976	(39.3)
90+	797	(3.9)	335	(6.7)
*Sex*				
Male	12,001	(59.1)	3288	(65.3)
Female	8308	(40.9)	1744	(34.7)
*Ethnicity*				
White	16,601	(81.7)	4225	(84.0)
Asian	882	(4.3)	216	(4.3)
Black	209	(1.0)	51	(1.0)
Mixed	41	(0.2)	11	(0.2)
Other	478	(2.35)	74	(1.5)
Unknown	2098	(10.3)	455	(9.0)
*IMD*				
1 (least deprived)	4523	(22.3)	988	(19.6)
2	4348	(21.4)	1095	(21.8)
3	924	(19.3)	951	(18.9)
4	3729	(18.4)	1002	(20.0)
5 (most deprived)	3762	(18.5)	989	(19.7)
Unknown	23	(0.1)	7	(0.1)
*BMI*				
< 18.5	690	(3.4)	113	(2.2)
18.5–24.9	5585	(27.5)	1241	(24.7)
25.0–29.9	6202	(30.5)	1652	(32.8)
> 30	4248	(20.9)	1329	(26.4)
Unknown	3584	(17.6)	697	(13.9)
*Smoking*				
Non-smoker	3095	(15.2)	661	(13.1)
Ex-smoker	14,123	(69.5)	3763	(74.8)
Current smoker	3047	(15.0)	600	(11.9)
Unknown	44	(0.2)	8	(0.2)
*Alcohol*				
Non-drinker	2714	(13.4)	708	(14.1)
Ex-drinker	252	(1.2)	80	(1.6)
Current drinker	11,467	(56.5)	3077	(61.1)
Unknown	5876	(28.9)	1167	(23.2)
*Comorbidities*				
Hypertension	9709	(47.8)	3027	(60.2)
COPD	4501	(22.2)	1367	(27.2)
IHD	4297	(21.2)	2496	(49.6)
CKD	4518	(22.2)	1980	(39.3)
Type II diabetes	4152	(20.4)	1450	(28.9)
Anaemia	3359	(16.5)	1193	(23.7)
Atrial fibrillation	1964	(9.7)	1619	(32.2)
Stroke	2120	(10.4)	769	(15.3)
Valvular heart disease	1188	(5.8)	963	(19.1)
Peripheral arterial disease	1064	(5.2)	493	(9.8)
*Medications*				
Statin	9501	(46.8)	3234	(64.3)
Antiplatelets	6989	(34.4)	2604	(51.7)
ACE inhibitors	4913	(24.2)	2255	(44.8)
Calcium-channel blockers	5154	(25.4)	1417	(28.2)
Beta-blockers	4332	(21.3)	2491	(49.5)
Diuretics	2905	(14.3)	955	(19.0)
ARBs	3020	(14.9)	1218	(24.2)
Anticoagulants	1857	(9.1)	1387	(27.6)
Short-acting nitrates	1426	(7.0)	796	(15.8)
Long-acting nitrates	1132	(5.6)	762	(15.1)
Digoxin	592	(2.9)	588	(11.7)
MRAs	364	(1.8)	725	(14.4)
Amiodarone	159	(0.8)	213	(4.2)
Ivabradine	58	(0.3)	76	(1.5)
ARNI	0		19	(0.4)

IPF-CS: idiopathic pulmonary fibrosis clinical syndrome; HF: heart failure; IMD: index of multiple deprivation; BMI: body mass index; COPD: chronic obstructive pulmonary disease; IHD: ischaemic heart disease; CKD: chronic kidney disease; ACEi: angiotensin converting enzyme inhibitors; ARBs: angiotensin receptor blockers; MRAs: mineralocorticoid receptor antagonists; ARNI: angiotensin receptor neprilysin inhibitor

### Temporal trends in left-sided HF incidence, 2010–2019

**Fig. 1:**
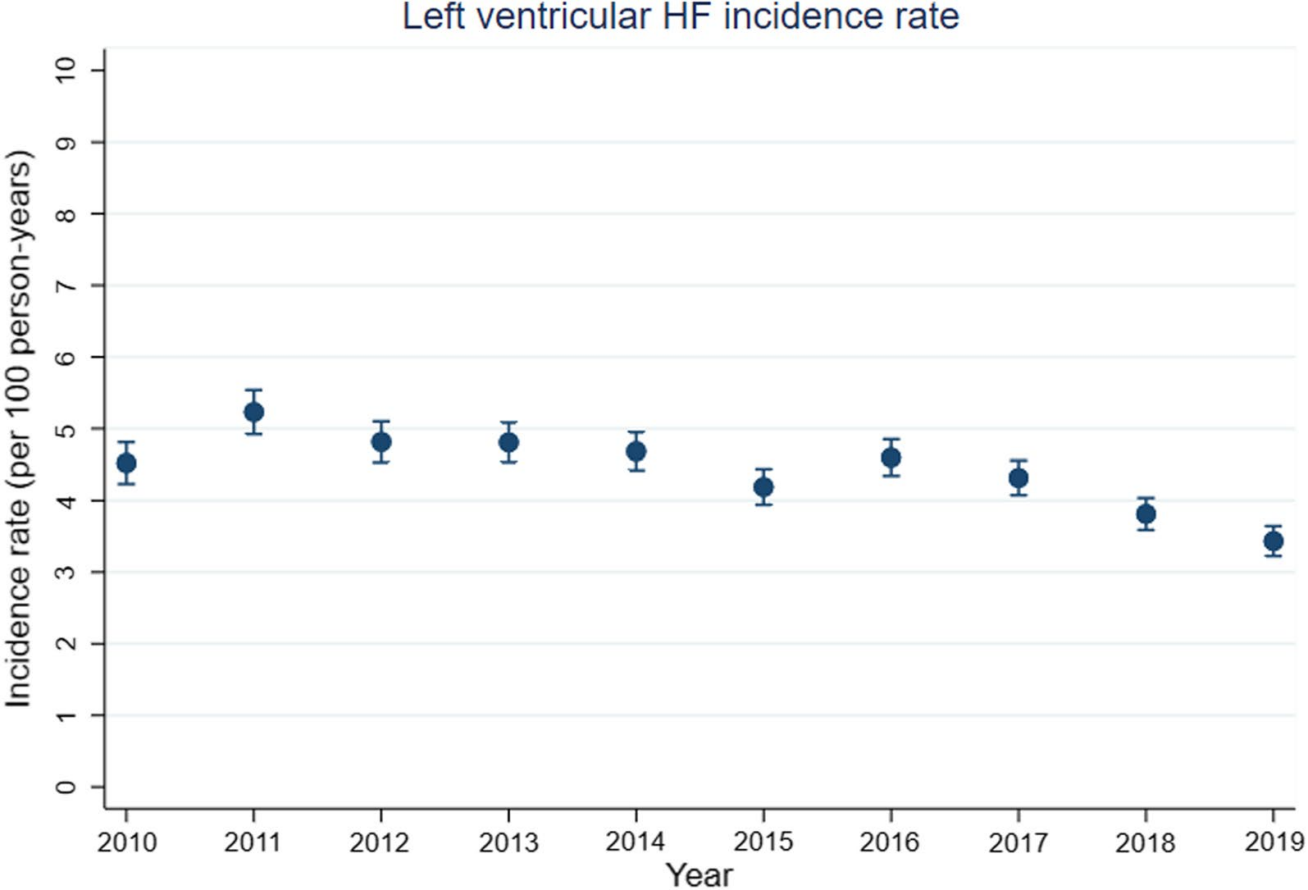
Annual crude incidence rate per 100 person-years of left ventricular heart failure (HF) in patients with IPF-CS between 2010 and 2019. Vertical bars for each estimate represent 95% confidence intervals

Left-sided HF incidence rate per 100 person-years (95% CI) remained relatively stable between 2010–2017 but decreased modestly from 4.3 (3.9–4.8) in 2017 to 3.4 (3.0–3.9) in 2019 (Fig. 1, Additional file 1: Table S6). Although a similar trend was noted in both sexes and different age groups, at all time-points the incidence rate was higher in men than women (2019: 4.05 (3.49–4.69) in men; 2.62 (2.1–3.22) in women) (Additional file 1: Tables S7-S8 and Fig. S9) and higher with increasing age (2019: 1.42 (0.61–2.79) in those aged 40–59, 2.62 (2.15–3.15) in those aged 60–79 and 4.93 (4.18–5.78) in those aged ≥ 80 (Additional file 1: Tables S10-S12 and Fig. S13). This equates to an almost twofold increase in left-sided HF incidence rate per 20-year increase in age.

### Temporal trends in left-sided HF prevalence, 2010–2019

**Fig. 2:**
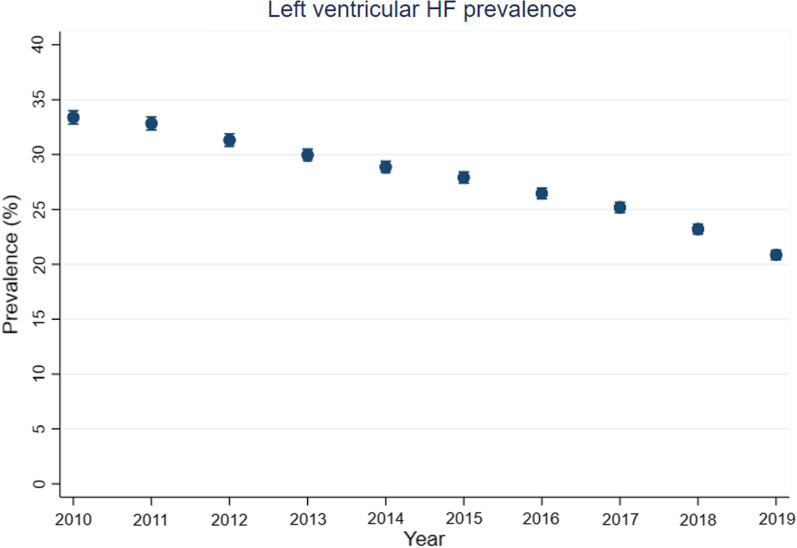
Annual prevalence (%) of left ventricular heart failure (HF) in patients with IPF-CS between 2010 and 2019. Vertical bars for each estimate represent 95% confidence intervals

Between 2010 and 2019, left-sided HF prevalence decreased from 33.4% (95% CI 32.2–34.6) in 2010 to 20.9% (20.0–21.7) in 2019 (Fig. 2, Additional file 1: Table S14). At all time-points, prevalence was higher in men than women (Additional file 1: Tables S15-S16 and Fig. S17), and with increasing age (Additional file 1: Tables S18-S20 and Fig. S21).

### Sensitivity analyses

Left-sided HF incidence and prevalence estimates remained relatively robust to various sensitivity analyses. Varying IPF-CS definitions resulted in HF prevalence estimates which were 0.1–2.5% lower in 2019 than the main analysis (Additional file 1: Fig. S28 and Tables S29-S31). By contrast, exclusion of IPF-CS patients diagnosed younger than age 50 or 60 resulted in 0.64% and 2.28% higher estimates in 2019, respectively (Additional file 1: Tables S32–S33). For incidence analyses, varying IPF-CS definitions resulted in estimates which were 0.24–1.02 cases per 100 person-years lower than in the main analysis (Additional file 1: Fig. S22 and tables S23–S27).

### Association between comorbid left-sided HF and mortality

**Fig. 3:**
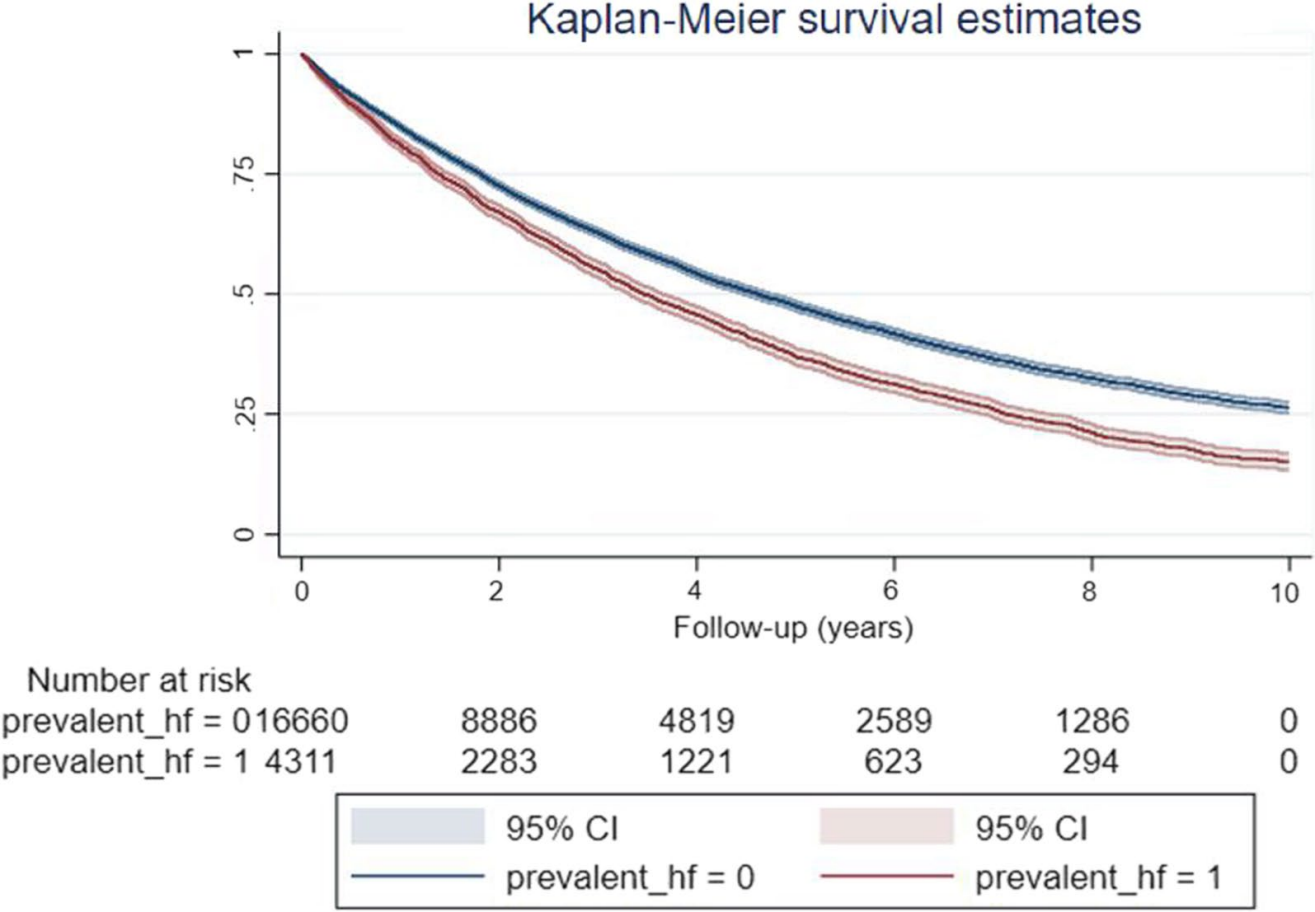
Kaplan–Meier survivor curve with 95% confidence intervals (CIs) comparing patients with IPF-CS with (prevalent_hf = 1) and without (prevalent_hf = 0) prevalent heart failure over 10 years of follow-up

20, 971 patients were included in the mortality analyses. Comorbid IPF-CS and prevalent left-sided HF was associated with poorer survival (Fig. 3), with an 8% increased risk of all-cause and 32% increased risk of cardiovascular mortality, following adjustment for age, gender, smoking status, BMI group, AF, IHD, hypertension, valve disease, COPD, diabetes and anaemia (Table 2). There was no significant difference in IPF-specific mortality risk between IPF-CS patients with and without left-sided HF.

**Table 2:** Crude and adjusted hazard ratios for the association between prevalent left-sided HF and risk of all-cause, cardiovascular and IPF-specific mortality

Mortality	Crude HR (N = 20,971)	95% CI	Adjusted HR (N = 20,971)	95%CI
All-cause	1.32	1.26–1.38	1.08*	1.03–1.14
Cardiovascular	2.59	2.20–3.04	1.32*	1.09–1.59
IPF-specific	1.06	0.99–1.14	0.92**	0.86–0.99

HR: hazard ratio; CI: confidence interval; HF: heart failure; IPF: idiopathic pulmonary fibrosis; BMI: body mass index; AF, atrial fibrillation; IHD: ischaemic heart disease; COPD: chronic obstructive pulmonary disease

*Adjusted for age (per 1 year increment), gender, smoking status, BMI group, AF, IHD, hypertension, valve disease, COPD, diabetes, anaemia

**Adjusted for age (per 1 year increment), gender, smoking status, BMI group, COPD

Multivariate models of factors influencing this association showed being older, male and having co-existing AF, IHD, diabetes or anaemia were associated with an increased risk of both all-cause and cardiovascular mortality (Additional file 1: Table S34). The proportional hazards assumption was met for both models (Additional file 1: Fig. S37), with no collinearity in either model.

In a sensitivity analysis of 18,796 patients where ethnicity and IMD were included in each model, only a modest 2% increase in adjusted risk for both all-cause and cardiovascular mortality was noted (Additional file 1: Table S35).

## Discussion

While previous studies have shown temporal trends in both incidence and prevalence of HF in the general population and in those with other chronic respiratory conditions such as COPD, to our knowledge, this is the first European study to use nationally-representative population-level data to explore temporal patterns of the burden of left-sided HF in IPF and adds to the growing body of evidence of the much higher burden of cardiovascular disease in patients with IPF than those in the general population and patients with COPD. Although left-sided HF incidence and prevalence in IPF patients in England is decreasing, our study shows that IPF patients are fourfold more likely to develop left-sided (HF) than COPD patients [ref] and having comorbid HF is poor prognostic marker which increases risk of both all-cause and cardiovascular mortality, with a comparatively higher risk of the former than the latter.

The modest reduction in HF incidence could reflect improved management of cardiovascular risk factors over time. Indeed, prescriptions for CVDs increased by 78% in England between 1991 and 2014 [38]. By contrast, decreasing prevalence of left-sided HF may be driven by high mortality of IPF patients, particularly as their historic median survival is 3–4 years post diagnosis [1], mortality has been shown to be rising [39], and comorbid HF confers an even greater mortality risk. However, an alternative explanation could be a relative decrease in prevalence driven by an increasing denominator relative to the numerator. While the absolute number of prevalent HF cases increased annually from 2010 to 2014 in our study, the total number of IPF-CS patients increased relatively more, in-keeping with evidence that IPF incidence is increasing in the UK [18]. Coupled with decreasing HF incidence in more recent years and high mortality in those with comorbid HF, there may be fewer patients with IPF who develop left-sided HF. Those who do have poor survival and in the context of rising numbers of IPF patients in the UK, this may be driving a relative reduction in the proportion of patients living with comorbid IPF and left-sided HF in more recent years.

We were unable to ascertain from the data whether IPF patients with HF had HFrEF or HFpEF, although this would be useful to characterise. While HF prevalence has been noted to be increasing globally, many studies show a stable or decreasing HF incidence. An increasingly ageing population and improved HF survival due to treatment advances may explain increased prevalence while better treatment of acute coronary syndromes due to the advent of revascularisation therapies may explain reduced incidence of HF [20]. Furthermore, the risk factors for HFpEF are different to those for HFrEF, with treatment of the former relying on treating underlying risk factors. This may explain why the incidence of HFrEF is decreasing while the incidence of HFpEF is increasing.

As discussed earlier, the treatment landscape of IPF has changed significantly in the last decade and it is unclear how these changing treatment patterns have influenced the burden of comorbidities such as left-sided HF in IPF. As antifibrotics are prescribed in tertiary care, we did not have access to antifibrotic use data in CPRD Aurum and therefore were unable to explore this potential association. Interestingly however, antifibrotics have been shown to have cardioprotective properties within the context of HFpEF, albeit this has not been explored specifically in the context of comorbid IPF and HF [28, 29]. To lend further support to a possible association between the use of antifibrotics and the modest reduction left-sided HF incidence noted in this study, pirfenidone was licensed in the UK by NICE (National Institute of Clinical Excellence) in 2013 and nintedanib in 2016 and it is interesting to note in our data that it is more from 2017 onwards that a sustained reduction in the left-sided HF incidence rate is noted (Fig. 1, Additional file 1: Table S6). However, any potential cardioprotective effect of antifibrotics may be counteracted by the fact that only a proportion of IPF patients receive these treatments. An analysis of IPF patients from the British Thoracic Society (BTS) ILD registry between 2013 and 2019 showed that although more patients were prescribed anti-fibrotic therapies during this period, 43% of patients were ineligible for treatment based upon NICE prescribing criteria [7] and 26% of patients discontinued the drugs due to treatment-associated adverse effects [39]. The introduction of antifibrotics therefore is unlikely to fully explain the modest reduction in left-sided HF incidence noted in England during the study period. The underlying explanation is more likely multifactorial, including increased screening and treatment for cardiovascular risk factors in clinical practice. Interestingly, both in vitro and in vivo studies have shown some antifibrotic properties of commonly used cardiovascular medications including ACE inhibitors, angiotensin receptor antagonists, mineralocorticoid receptor antagonists and angiotensin receptor neprilysin inhibitors [40, 41], although this has rarely translated to improved outcomes in clinical trials. A limitation of the study however is that use of cardiovascular medications was not adjusted for in the mortality analyses, with this necessitating future work to characterise further any potential modulation of cause-specific mortality risk by cardiovascular medication use in patients with IPF and comorbid left-sided HF.

Our HF incidence estimates are significantly lower than those reported by a US study, the only other population-based study which has explored the burden of left-sided HF in an IPF patient population. Using claims data between 2001 and 2008 for over 9000 patients, this study reported an incidence rate of 67.5 cases per 1000 person-years [23]. Given temporal changes in left-sided HF incidence exist, the different follow-up periods between the studies may explain our lower estimates.

By contrast, our prevalence estimates are higher than those from smaller cohort studies which range from 8 to 23% [12, 23–26]. This could be due to differences in IPF and HF case ascertainment, study periods and follow-up time, and sample sizes. For instance, while the US study discussed above noted a prevalence of 20% [23], an analysis of data from a US single tertiary centre showed a lower estimate of 11% [24]. Given published estimates derive from cross-sectional studies and temporal patterns in HF prevalence may exist, comparison of estimates between studies is challenging.

Consistent with other studies, male gender was found to be a significant risk factor for having a higher HF burden [21]. Although reasons for this are unclear, possible explanations include higher HF risk factor burden in men than women or different healthcare seeking behaviours between genders. Furthermore, male sex and older age have been recognised as risk factors for delayed IPF diagnosis [27], and this diagnostic delay may contribute to worse IPF disease severity at diagnosis in older men, with higher consequent risk of HF.

Based on our estimates, left-sided HF prevalence is approximately 30-fold higher and incidence is almost 12-fold higher in IPF patients than in the general UK population [12]. Comparison of HF prevalence estimates between IPF and COPD patients is more challenging due to heterogeneity in published estimates, with estimates ranging between 5 and 41% in different COPD cohorts [28]. Assuming that estimates for community-based populations lie towards the lower end of this range, left-sided HF prevalence may be considerably higher in IPF than COPD patients. More comparable data are available on HF incidence in COPD, with a UK study using CPRD data showing a crude incidence rate of 1.18 cases per 100 person-years [29]. This is notably 3.9 times lower than in our IPF cohort at comparable timepoints.

Our all-cause mortality estimates are in-keeping with two studies showing an increased mortality risk with comorbid IPF and HF, although our estimates are 29% lower than those in an Italian study [21], and threefold lower than those from a smaller Japanese study [13]. Direct comparison is however challenging due to adjustment for different confounders in the former and presentation of unadjusted estimates in the latter.

While this study cannot explain why left-sided HF burden is so high in IPF relative to COPD patients, there are several possible theories. Shared risk factors such as smoking and ageing or shared pathological mechanisms such as telomere dysfunction and molecular and cellular fibrosis in both IPF and HF may contribute [30, 31]. Alternatively, a higher burden of HF risk factors such as IHD in IPF patients has been noted [5, 7, 32]. Furthermore, pulmonary hypertension is highly prevalent in IPF patients [11], and causes an enlarged overloaded right ventricle, which may drive LV diastolic dysfunction due to ventricular interdependence [9]. While there is biological plausibility for the common co-existence of left-sided HF with IPF, estimates of its burden from observational studies could nevertheless be confounded by a high rate of misdiagnosis of IPF as HF, as both share common symptoms such as dyspnoea and cough [33]. Indeed, 57.4% of HF were diagnosed pre IPF-CS in this cohort, suggesting misdiagnosis of non-specific symptoms such as dyspnoea perhaps by primary care physicians.

Strengths of this study include use of nationally representative population-level data linked to national mortality and secondary care data to explore left-sided HF epidemiology in IPF patients. This contrasts previous studies to date which have largely explored cohorts from single centres or registries. Although there is recognised heterogeneity in IPF coding terms in electronic healthcare records, left-sided HF incidence and prevalence estimates remained robust despite several sensitivity analyses optimising IPF case ascertainment. While there is potential risk of case misclassification due to dependency on codes entered in primary care, we draw confidence in the validity of ascertainment of both our exposure and outcome due to high positive predictive values (82–95%) of both HF and IPF diagnoses in primary care records [17, 34].

Nevertheless, this study has several limitations. The cohort was predominantly Caucasian with patients residing in England, limiting generalisability of findings to other populations. Almost 80% of patients in the cohort also had non-specific codes for pulmonary fibrosis and although we used proxy measures in the sensitivity analyses to optimise IPF-CS case ascertainment such as exclusion of those with evidence of a potential non-idiopathic cause for pulmonary fibrosis based on mortality data, there is nonetheless a possibility that some cases were misclassified. A more comprehensive study of the validity of IPF codes in CPRD Aurum is currently in progress and would inform confidence in the results from this study. Furthermore, we did not have access to imaging data to determine IPF disease severity. This is important to ascertain as studies have shown IPF patients with a rapid disease course were more likely to be diagnosed with HF post IPF diagnosis [42]. Furthermore, we were unable to establish from the data available whether patients were on antifibrotic therapies. This would have been useful to ascertain as IPF disease severity likely influences HF risk and both all-cause and cause-specific mortality. In addition, most patients with comorbid left-sided HF also had non-specific HF codes, and there is a risk that patients with right-sided or biventricular HF were coded as such, which may have caused overestimation of HF incidence or prevalence, however based on ongoing work we think this is unlikely. Furthermore, we were unable to establish HF phenotypes from the data available and whether the high burden of HF was due to HFrEF or HFpEF and whether these phenotypes have changed with time. This is important to establish as HFrEF has been associated with a higher mortality risk in observational studies in a non-IPF context [43]. Furthermore, there was some overlap in the terms used to identify incident and prevalent HF in the codelists, which may have also biased the estimates of HF incidence and prevalence reported. Due to the absence of echocardiographic data, we were also unable to determine HF disease severity. Use of HF medications was also not explored in mortality analyses, which may have affected the estimates reported.

Lastly, there was a significant proportion of missing data for alcohol intake, ethnicity and BMI. As missing data for these variables has been shown to not be missing at random in CPRD Aurum, we conducted a complete-case mortality analysis, but acknowledge this may have introduced some bias. For instance, patients with missing alcohol or BMI data are more likely to not have engaged with healthcare providers as often and by extension may have been less compliant with their medications or monitoring investigations for their IPF or HF. Therefore, it is possible that the patients excluded from the mortality analyses due to missing data may have generally been less healthy and may have had more severe disease severity of both their IPF and HF. The exclusion of these patients due to missing data may have therefore underestimated the risk of all-cause and cause-specific mortality in those patients with comorbid IPF and HF.

## Conclusion

This study shows temporal changes in both incidence and prevalence of left-sided HF in IPF patients in England, with modest reduction in incidence possibly reflecting improved screening and management of cardiovascular risk factors. However, despite decreasing incidence and prevalence, left-sided HF burden is considerably higher in IPF patients than in patients with other comparable chronic respiratory diseases and confers a higher mortality risk. Further studies are needed to explore HF phenotypes in IPF patients, whether there is a temporal relationship between the two diseases, what the aetiological mechanisms driving this association are, what drives the noted gender differences and whether HF treatment modifies the mortality risk.

## Supplementary Information

**Additional file 1.**Supplementary material.

## Data Availability

Data are available on request from the CPRD. Their provision requires the purchase of a license, and this license does not permit the authors to make them publicly available to all. This work used data from the version collected in November 2020 and have clearly specified the data selected in the Methods section. To allow identical data to be obtained by others, via the purchase of a license, the code lists have been provided on GitHub. Licenses are available from the CPRD (http://www.cprd.com): The Clinical Practice Research Datalink Group, The Medicines and Healthcare products Regulatory Agency, 10 South Colonnade, Canary Wharf, London E14 4PU. This study used existing data from the UK CPRD electronic health record database, this data resource is accessible only to researchers with protocols approved by the CPRD’s independent scientific advisory committee; therefore, no additional unpublished data are available. All data management and analysis computer code are available on request. The study protocol and analysis plan are available in the associated Additional file 1.

## Abbreviations

ACEi: Angiotensin converting enzyme inhibitors
AF: Atrial fibrillation
ARBs: Angiotensin receptor blockers
ARNI: Angiotensin receptor neprilysin inhibitor
ATS: American Thoracic Society
ALAT: Latin American Thoracic Association
BMI: Body mass index
CI: Confidence interval
CKD: Chronic kidney disease
COPD: Chronic obstructive pulmonary disease
CPRD: Clinical Practice Research Datalink
CVD: Cardiovascular disease
DAG: Directed acyclic graph
EHR: Electronic healthcare records
ERS: European Respiratory Society
HES: Hospital Episode Statistics
HF: Heart failure
HFrEF: Heart failure with reduced ejection fraction
HFpEF: Heart failure with preserved ejection fraction
HR: Hazard ratio
HRA: Health Research Authority
ICD-10: International classification of diseases-10
IHD: Ischaemic heart disease
IMD: Index of Multiple Deprivation
IPF: Idiopathic pulmonary fibrosis
IPF-CS: Idiopathic pulmonary fibrosis clinical syndrome
IQR: Interquartile range
ISAC: Independent Scientific Advisory Committee
JRS: Japanese Respiratory Society
MHRA: Medicines and Healthcare Products Regulatory Agency
MRA: Mineralocorticoid receptor antagonists
LV: Left ventricular
NHS: National Healthcare Service
ONS: Office of National Statistics
SD: Standard deviation
UK: United Kingdom
US: United States
