# Supplementary material for: Left-sided heart failure burden and mortality in idiopathic pulmonary fibrosis: a population-based study
Source: BMC Pulm Med. 2022 May 12;22:190. doi: 10.1186/s12890-022-01973-5 (PMC9097426; doi:10.1186/s12890-022-01973-5)

APPENDIX

Table of Contents **Page**

Fig S1: Directed Acyclic Graph to identify confounders in association between comorbid HF and risk of all-cause mortality in IPF-CS patients……………………………………………………………....4

Fig S2: Directed Acyclic Graph to identify confounders in association between comorbid HF and risk of cardiovascular mortality in IPF-CS patients…………………………………………………..........4

Fig S3: Directed Acyclic Graph to identify confounders in association between comorbid HF and risk of IPF mortality in IPF-CS patients………………………………………………..…...................5

Table S4: Distribution of regions in England in which IPC-CS patients included in the cohort were residing…….5

Table S5: Distribution of IPF-CS diagnostic terms in the cohort…………………………………………6

Table S6: Incidence rate of Left HF per 100 person-years (95%CI) for each year of the study period in IPF-CS patients……………………………………………………………………………………………….6

Table S7: Incidence rate of Left HF per 100 person-years (95%CI) for each year of the study period in men with IPF-CS……………………………………………………………………………………………6

Table S8: Incidence rate of Left HF per 100 person-years (95%CI) for each year of the study period in women with IPF-CS……………………………………………………………………………………….7

Fig S9: Annual crude incidence rate per 100 person-years of left ventricular heart failure (HF) in patients with IPF-CS between 2010 – 2019, stratified by sex…………………………………….7

Table S10: Incidence rate of Left HF per 100 person-years (95%CI) for each year of the study period in those with IPF-CS aged 40-59 years old…………………………………………………………….8

Table S11: Incidence rate of Left HF per 100 person-years (95%CI) for each year of the study period in those with IPF-CS aged 60-79 years old…………………………………………………………….8

Table S12: Incidence rate of Left HF per 100 person-years (95%CI) for each year of the study period in those with IPF-CS aged 80 years and over………………………………………………………..8

Fig S13: Annual incidence rate per 100 person-years of left ventricular heart failure (HF) in patients with IPF-CS between 2010 – 2019 stratified by age…………………………………………….9

Table S14: Prevalence of Left HF (%) (95%CI) for each year of the study period…………………10

Table S15: Prevalence of Left HF (%) (95%CI) for each year of the study period in men with IPF-CS……10

Table S16: Prevalence of Left HF (%) (95%CI) for each year of the study period in women with IPF-CS……10

Fig S17: Annual prevalence (%) of left ventricular heart failure (HF) in patients with IPF-CS between 2010 – 2019 stratified by sex………………………………………………………………………….11

Table S18: Prevalence of Left HF (%) (95%CI) for each year of the study period in those with IPF-CS aged 40-59 years……………………………………………………………………………………………..11

Table S19: Prevalence of Left HF (%) (95%CI) for each year of the study period in those with IPF-CS aged 60-79 years……………………………………………………………………………………………..11

Table S20: Prevalence of Left HF (%) (95%CI) for each year of the study period in those with IPF-CS aged 80 years and over……………………………………………………………………………………12

Fig S21: Annual prevalence (%) of left ventricular heart failure (HF) in patients with IPF-CS between 2010 – 2019 stratified by age…………………………………………………………………………13

Fig S22: Sensitivity Analyses - Left HF incidence**………………………………………………………….**14

Table S23: Incidence rate of Left HF per 100 person-years (95%CI) for each year of the study period when excluding those with non-specific IPF-CS codes……………………………………………..15

Table S24: Incidence rate of Left HF per 100 person-years (95%CI) for each year of the study period when excluding those with potential other causes for pulmonary fibrosis……………..15

Table S25: Incidence rate of Left HF per 100 person-years (95%CI) for each year of the study period when excluding those diagnosed with IPF-CS post change of diagnostic guidelines in 2018……15

Table S26: Incidence rate of Left HF per 100 person-years (95%CI) for each year of the study period when excluding those diagnosed with IPF-CS younger than 50 years old…………………16

Table S27: Incidence rate of Left HF per 100 person-years (95%CI) for each year of the study period when excluding those diagnosed with IPF-CS younger than 60 years old…………………16

Fig S28: Sensitivity Analyses - Left HF prevalence……………………………………………………….17

Table S29: Prevalence of Left HF (%) (95%CI) in those with IPF-CS for each year of the study period when excluding those with non-specific IPF-CS codes……………………………………………..18

Table S30: Prevalence of Left HF (%) (95%CI) in those with IPF-CS for each year of the study period when excluding those with potential other causes for pulmonary fibrosis……………..18

Table S31: Prevalence of Left HF (%) (95%CI) in those with IPF-CS for each year of the study period when excluding those diagnosed with IPF-CS post change of diagnostic guidelines in 2018……18

Table S32: Prevalence of Left HF (%) (95%CI) in those with IPF-CS for each year of the study period when excluding those diagnosed with IPF-CS when younger than 50 years old……….19

Table S33: Prevalence of Left HF (%) (95%CI) in those with IPF-CS for each year of the study period when excluding those diagnosed with IPF-CS when younger than 60 years old……….19

Table S34: Multivariate models of the association between prevalent heart failure and risk of all-cause and cardiovascular mortality in IPF-CS……………………………………………………………20

Table S35: Multivariate models of the association between prevalent heart failure and risk of all-cause and cardiovascular mortality in IPF-CS: inclusion of ethnicity and IMD in the model as a sensitivity analysis……………………………………………………………………………………………………..21

Table S36: Multivariate models of the association between prevalent heart failure and risk of IPF-specific mortality in IPF-CS…………………………………………………………………………………………22

Fig S37: Graphical assessment of whether proportional hazards assumption met in each multivariate Cox model in main analysis……………………………………………………………………………23

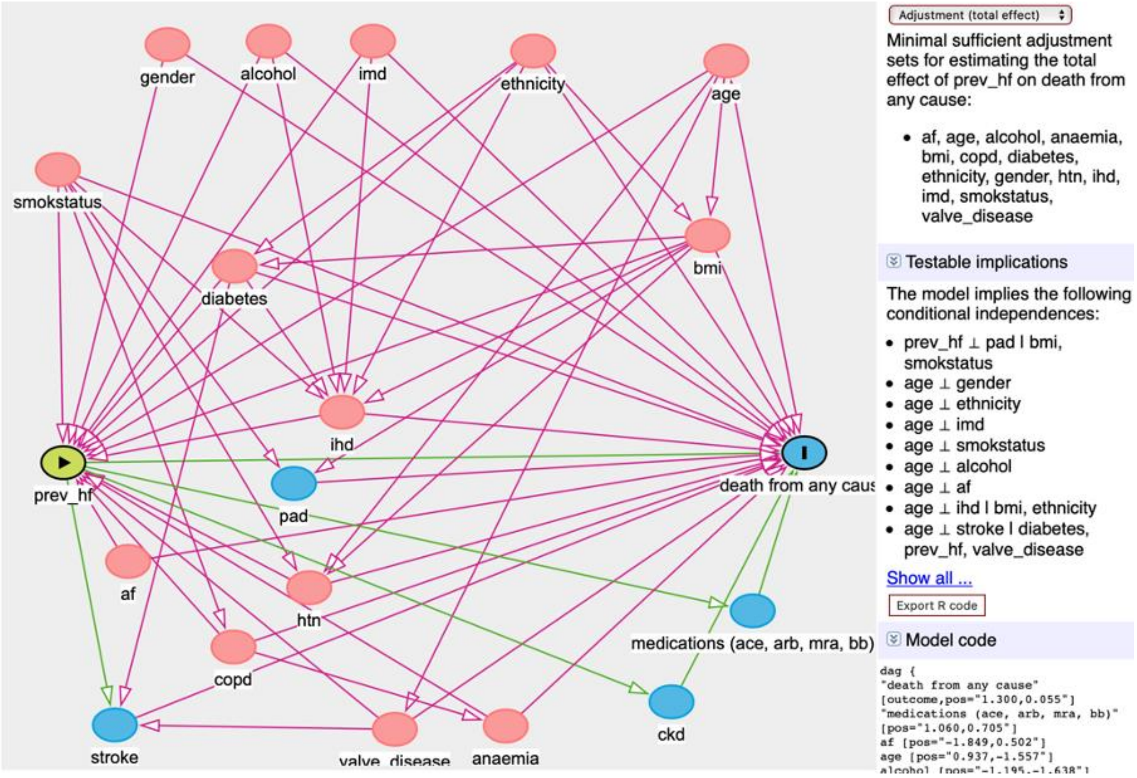

Fig S1: Directed Acyclic Graph (DAG) used to identify possible confounders in the association between comorbid HF and risk of all-cause mortality. DAG drawn using daggity.net web resource.

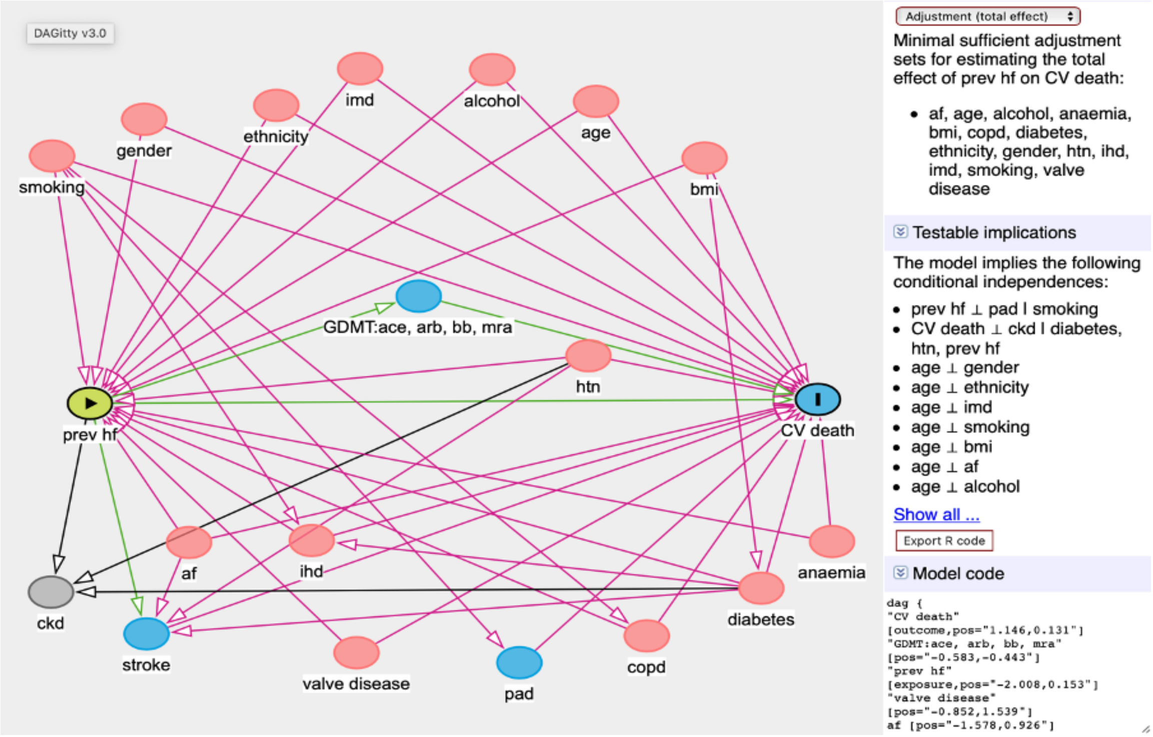

Fig S2: Directed Acyclic Graph (DAG) used to identify possible confounders in the association between comorbid HF and risk of cardiovascular mortality. DAG drawn using daggity.net web resource.

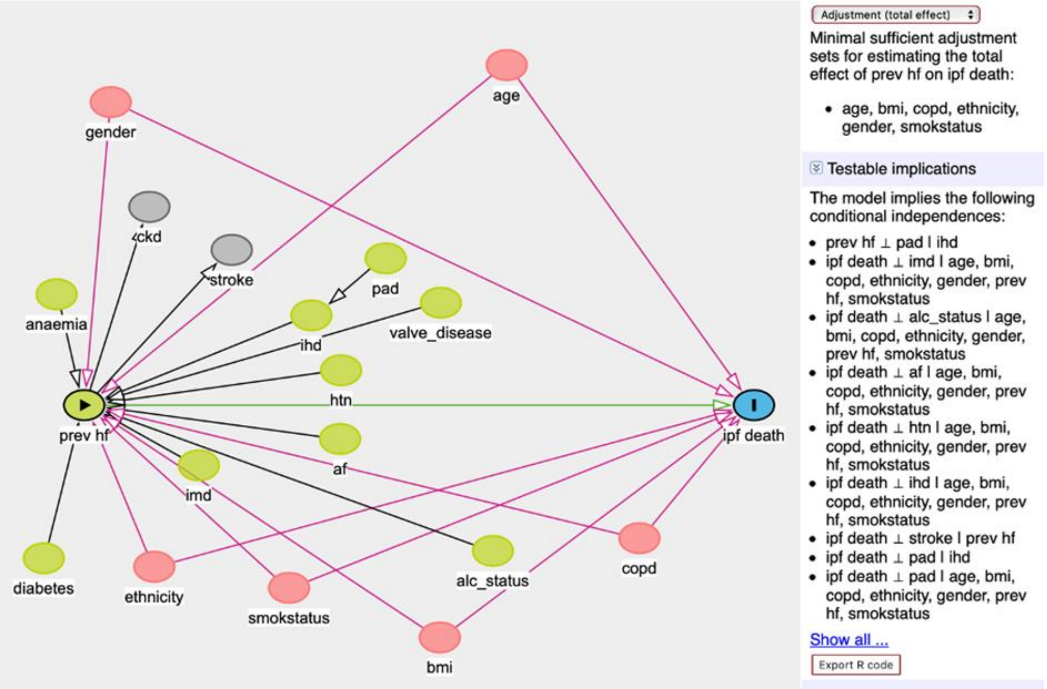

Fig S3: Directed Acyclic Graph (DAG) used to identify possible confounders in the association between comorbid HF and risk of IPF mortality. DAG drawn using daggity.net web resource.

Table S4: Distribution of regions in England in which IPF-CS patients included in the cohort were residing

Region	Number of patients (%)
North West	5790 (22.9)
West Midlands	4374 (17.3)
South West	3219 (12.7)
South Central	2999 (11.8)
London	2898 (11.4)
South East Coast	2065 (8.2)
Yorkshire and the Humber	1201 (4.7)
North East	1103 (4.4)
East of England	1011 (4.0)
East Midlands	647 (2.6)
Unknown	34 (0.1)

Table S5: Distribution of IPF-CS diagnostic terms in the cohort

Term	Number of patients (%)
Pulmonary fibrosis	9357 (36.9)
Diffuse pulmonary fibrosis	7191 (28.4)
O/E – fibrosis of lung	2732 (10.8)
Idiopathic pulmonary fibrosis	2544 (10.0)
Idiopathic fibrosing alveolitis	1256 (5.0)
O/E – fibrosis of lung present	822 (3.2)
Usual interstitial pneumonitis	637 (2.5)
Cryptogenic fibrosing alveolitis	521 (2.1)
Idiopathic fibrosing alveolitis NOS	139 (0.6)
Fibrosis of lung	104 (0.4)
Idiopath. fibrosing alveolitis	29 (0.1)
Hamman – Rich syndrome	6 (0.02)
UIP – usual interstitial pneumonitis	<5 (0.01)

Table S6: Incidence rate of Left HF per 100 person-years (95%CI) for each year of the study period in IPF-CS patients

Year	Number of new HF cases	Person years	Incidence rate per 100 person-years (95% CI)
2010	241	5331.33	4.52 (3.97 – 5.13)
2011	290	5544.25	5.23 (4.65 – 5.87)
2012	286	5939.52	4.82 (4.27 – 5.41)
2013	307	6385.72	4.81 (4.28 – 5.38)
2014	311	6640.47	4.68 (4.18 – 5.23)
2015	285	6804.72	4.19 (3.72 – 4.70)
2016	327	7111.89	4.6 (4.11 – 5.12)
2017	321	7437.65	4.32 (3.86 – 4.81)
2018	293	7686.39	3.81 (3.39 – 4.27)
2019	271	7892.16	3.43 (3.04 – 3.87)

Table S7: Incidence rate of Left HF per 100 person-years (95%CI) for each year of the study period in men with IPF-CS

Year	Number of new HF cases	Person years	Incidence rate per 100 person-years (95% CI)
2010	165	3008.36	5.48 (4.68 – 6.39)
2011	189	3123.69	6.05 (5.22 – 6.98)
2012	190	3320.23	5.72 (4.94 – 6.60)
2013	195	3546.14	5.50 (4.75 – 6.33)
2014	197	3669.56	5.37 (4.64 – 6.17)
2015	189	3792.25	4.98 (4.30 – 5.75)
2016	203	3990.43	5.09 (4.41 – 5.84)
2017	205	4214.11	4.86 (4.22 – 5.58)
2018	192	4361.38	4.40 (3.80 – 5.07)
2019	182	4490.40	4.05 (3.49 – 4.69)

Table S8: Incidence rate of Left HF per 100 person-years (95%CI) for each year of the study period in women with IPF-CS

Year	Number of new HF cases	Person years	Incidence rate per 100 person-years (95% CI)
2010	76	2322.97	3.27 (2.58 – 4.09)
2011	101	2420.56	4.17 (3.40 – 5.07)
2012	96	2619.29	3.67 (2.97 – 4.48)
2013	112	2839.58	3.94 (3.25 – 4.75)
2014	114	2970.91	3.84 (3.17 – 4.61)
2015	96	3012.47	3.19 (2.58 – 3.89)
2016	124	3121.46	3.97 (3.30 – 4.74)
2017	116	3223.54	3.60 (2.97 – 4.32)
2018	101	3325.01	3.04 (2.47 – 3.69)
2019	89	3401.77	2.62 (2.10 – 3.22)

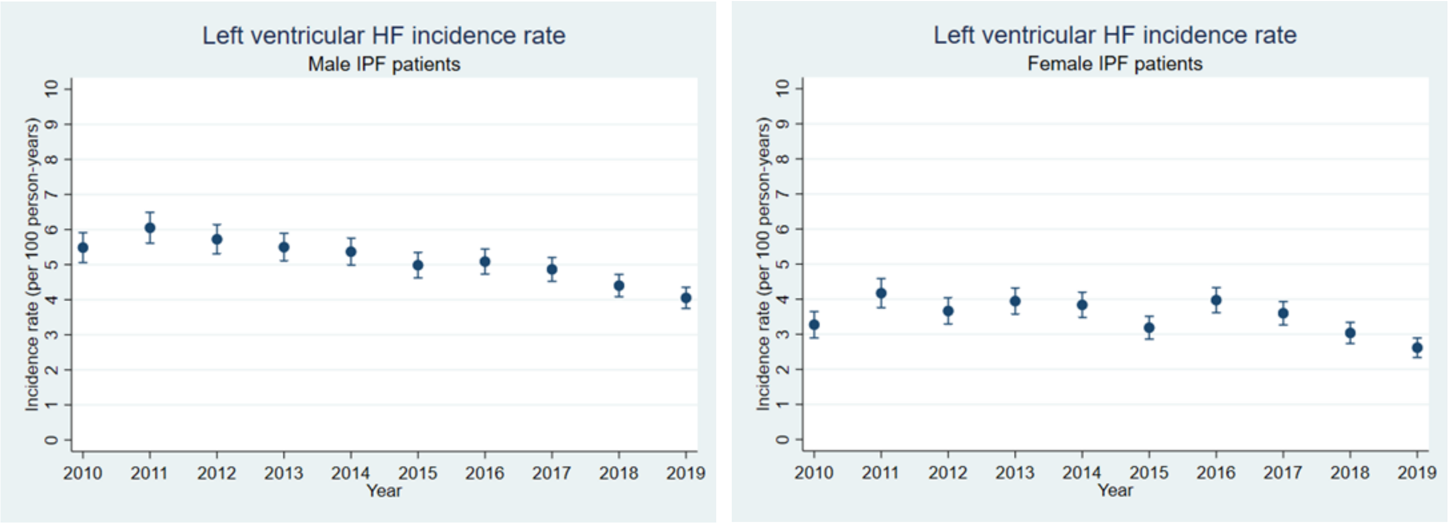

Fig S9: Annual incidence rate per 100 person-years of left ventricular heart failure (HF) in patients with IPF-CS between 2010 – 2019, stratified by sex. Incidence in men shown on the left and incidence in women on the right. Vertical bars for each estimate represent 95% confidence intervals.

Table S10: Incidence rate of Left HF per 100 person-years (95%CI) for each year of the study period in those with IPF-CS aged 40-59 years old

Year	Number of new HF cases	Person years	Incidence rate per 100 person-years (95% CI)
2010	2	493.35	0.41 (0.05 – 1.46)
2011	10	490.63	2.04 (0.98 – 3.75)
2012	11	536.64	2.05 (1.02 – 3.67)
2013	11	577.76	1.90 (0.95 – 3.41)
2014	11	567.75	1.94 (0.97 – 3.47)
2015	7	585.77	1.20 (0.48 – 2.46)
2016	6	601.60	1.00 (0.37 – 2.17)
2017	11	612.88	1.79 (0.90 – 3.21)
2018	13	588.34	2.21 (1.18 – 3.78)
2019	8	565.26	1.42 (0.61 – 2.79)

Table S11: Incidence rate of Left HF per 100 person-years (95%CI) for each year of the study period in those with IPF-CS aged 60-79 years old

Year	Number of new HF cases	Person years	Incidence rate per 100 person-years (95% CI)
2010	143	3250.36	4.40 (3.71 – 5.18)
2011	155	3345.36	4.63 (3.93 – 5.42)
2012	152	3466.48	4.38 (3.72 – 5.14)
2013	143	3681.72	3.88 (3.27 – 4.58)
2014	161	3857.07	4.17 (3.55 – 4.87)
2015	129	3890.54	3.32 (2.77 – 3.94)
2016	154	3957.40	3.89 (3.30 – 4.56)
2017	152	4067.46	3.74 (3.17 – 4.38)
2018	131	4171.06	3.14 (2.63 – 3.73)
2019	111	4244.03	2.62 (2.15 – 3.15)

Table S12: Incidence rate of Left HF per 100 person-years (95%CI) for each year of the study period in those with IPF-CS aged 80 years and over

Year	Number of new HF cases	Person years	Incidence rate per 100 person-years (95% CI)
2010	96	1587.63	6.05 (4.90 – 7.38)
2011	125	1708.26	7.32 (6.09 – 8.72)
2012	123	1936.40	6.35 (5.28 – 7.58)
2013	153	2126.24	7.20 (6.10 – 8.43)
2014	139	2215.65	6.27 (5.27 – 7.41)
2015	149	2328.41	6.40 (5.41 – 7.51)
2016	167	2552.88	6.54 (5.59 – 7.61)
2017	158	2757.31	5.73 (4.87 – 6.70)
2018	149	2926.99	5.09 (4.31 – 5.98)
2019	152	3082.88	4.93 (4.18 – 5.78)

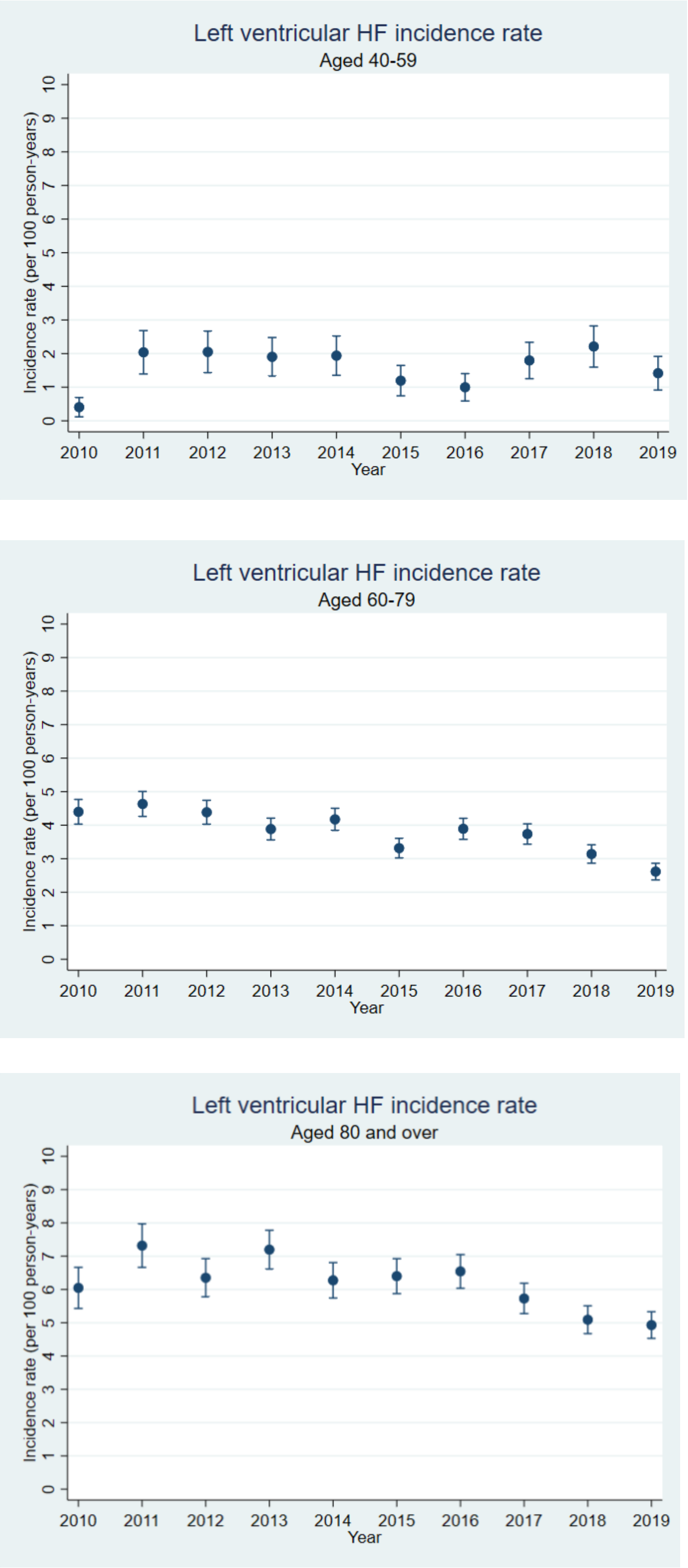

Fig S13: Annual incidence rate per 100 person-years of left ventricular heart failure (HF) in patients with IPF-CS between 2010 – 2019 stratified by age. Incidence in those aged 40-59 (A), 60-79 (B) and 80 and over (C). Vertical bars for each estimate represent 95% confidence intervals.

Table S14: Prevalence of Left HF (%) (95%CI) for each year of the study period

Year	Number of prevalent left HF cases	Total number of IPF patients	Prevalence (%) with 95% CI
2010	2022	6056	33.4 (32.2 – 34.6)
2011	2072	6310	32.8 (31.7 – 34.0)
2012	2111	6742	31.3 (30.2 – 32.4)
2013	2172	7250	30.0 (28.9 – 31.0)
2014	2181	7556	28.9 (27.8 – 29.9)
2015	2160	7741	27.9 (26.9 – 28.9)
2016	2156	8148	26.5 (25.5 – 27.4)
2017	2149	8533	25.2 (24.3 – 26.1)
2018	2044	8806	23.2 (22.3 – 24.1)
2019	1895	9087	20.9 (20.0 -21.7)

Table S15: Prevalence of Left HF (%) (95%CI) for each year of the study period in men with IPF-CS

Year	Number of prevalent left HF cases	Total number of IPF patients	Prevalence (%) with 95% CI
2010	1345	3465	38.8 (37.2 – 40.5)
2011	1393	3610	38.6 (37.0 – 40.2)
2012	1428	3848	37.1 (35.6 – 38.7)
2013	1459	4118	35.4 (34.0 – 36.9)
2014	1456	4279	34.0 (32.6 – 35.5)
2015	1448	4416	32.8 (31.4 – 34.2)
2016	1430	4685	30.5 (29.2 – 31.9)
2017	1407	4948	28.4 (27.2 – 29.7)
2018	1341	5091	26.3 (25.1 – 27.6)
2019	1231	5290	23.3 (22.1 – 24.4)

Table S16: Prevalence of Left HF (%) (95%CI) for each year of the study period in women with IPF-CS

Year	Number of prevalent left HF cases	Total number of IPF patients	Prevalence (%) with 95% CI
2010	677	2591	26.1 (24.5 – 27.9)
2011	679	2700	25.2 (23.5 – 26.8)
2012	683	2894	23.6 (22.1 – 25.2)
2013	713	3132	22.8 (21.3 – 24.3)
2014	725	3277	22.1 (20.7 – 23.6)
2015	712	3325	21.4 (20.0 – 22.9)
2016	726	3463	21.0 (19.6 – 22.4)
2017	742	3585	20.7 (19.4 – 22.1)
2018	703	3715	18.9 (17.7 – 20.2)
2019	664	3797	17.5 (16.3 – 18.7)

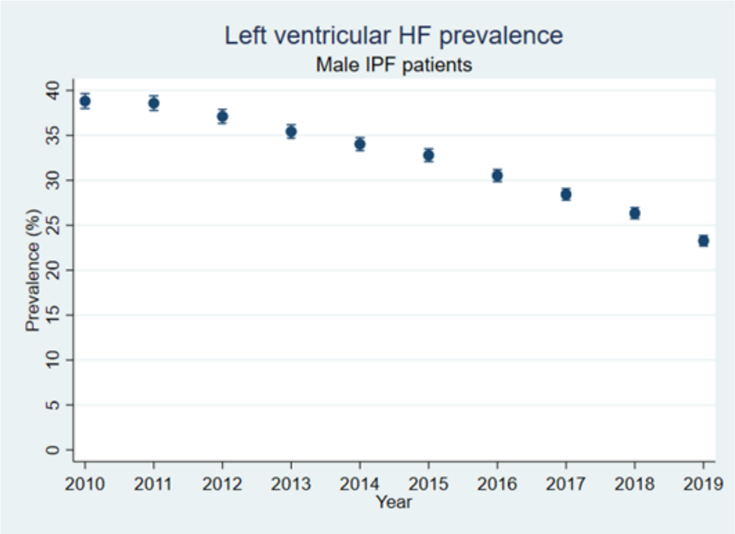

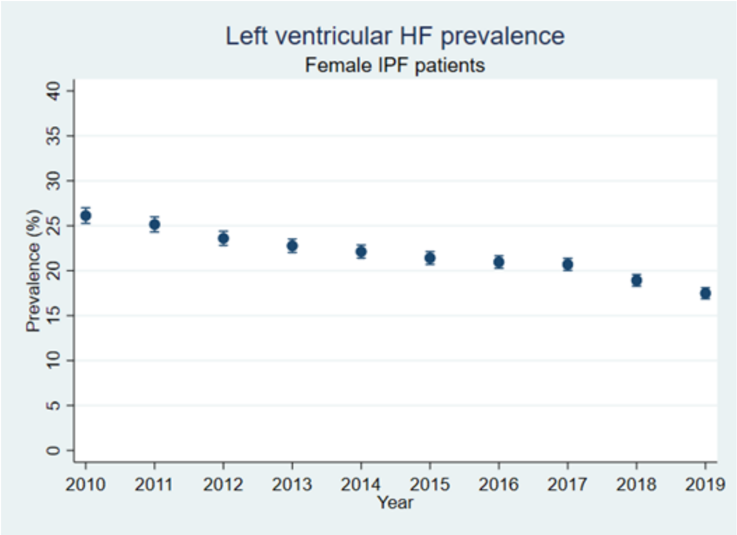

Fig S17: Annual prevalence (%) of left ventricular heart failure (HF) in patients with IPF-CS between 2010 – 2019 stratified by sex. Prevalence in men (left) and in women (right). Vertical bars for each prevalence estimate represent 95% confidence intervals.

Table S18: Prevalence of Left HF (%) (95%CI) for each year of the study period in those with IPF-CS aged 40-59 years

Year	Number of prevalent left HF cases	Total number of IPF patients	Prevalence (%) with 95% CI
2010	56	506	11.1 (8.5 – 14.1)
2011	61	510	12.0 (9.3 – 15.1)
2012	62	567	10.9 (8.5 – 13.8)
2013	62	602	10.3 (8.0 – 13.0)
2014	63	598	10.5 (8.2 – 13.3)
2015	57	612	9.3 (7.1 – 11.9)
2016	49	625	7.8 (5.9 – 10.2)
2017	46	643	7.2 (5.3 – 9.4)
2018	50	617	8.1 (6.1 – 10.5)
2019	49	595	8.2 (6.2 – 10.7)

Table S19: Prevalence of Left HF (%) (95%CI) for each year of the study period in those with IPF-CS aged 60-79 years

Year	Number of prevalent left HF cases	Total number of IPF patients	Prevalence (%) with 95% CI
2010	1112	3590	31.0 (29.5 – 32.5)
2011	1108	3724	29.8 (28.3 – 31.3)
2012	1096	3832	28.6 (27.2 – 30.1)

2013	1050	4082	25.7 (24.4 – 27.1)
2014	1007	4253	23.7 (22.4 – 25.0)
2015	956	4279	22.3 (21.1 – 23.6)
2016	936	4380	21.4 (20.2 – 22.6)
2017	909	4550	20.0 (18.8 – 21.2)
2018	857	4659	18.4 (17.3 – 19.5)
2019	767	4740	16.2 (15.1 – 17.3)

Table S20: Prevalence of Left HF (%) (95%CI) for each year of the study period in those with IPF-CS aged 80 years and over

Year	Number of prevalent left HF cases	Total number of IPF patients	Prevalence (%) with 95% CI
2010	854	1960	43.6 (41.4 –45.8)
2011	903	2076	43.5 (41.4 – 45.7)
2012	953	2343	40.7 (38.7 – 42.7)
2013	1060	2566	41.3 (39.4 – 43.2)
2014	1111	2705	41.1 (39.2 – 43.0)
2015	1147	2850	40.3 (38.4 – 42.1)
2016	1171	3143	37.3 (35.6 – 39.0)
2017	1194	3340	35.8 (34.1 – 37.4)
2018	1137	3530	32.2 (30.7 – 33.8)
2019	1079	3752	28.8 (27.3 – 30.2)

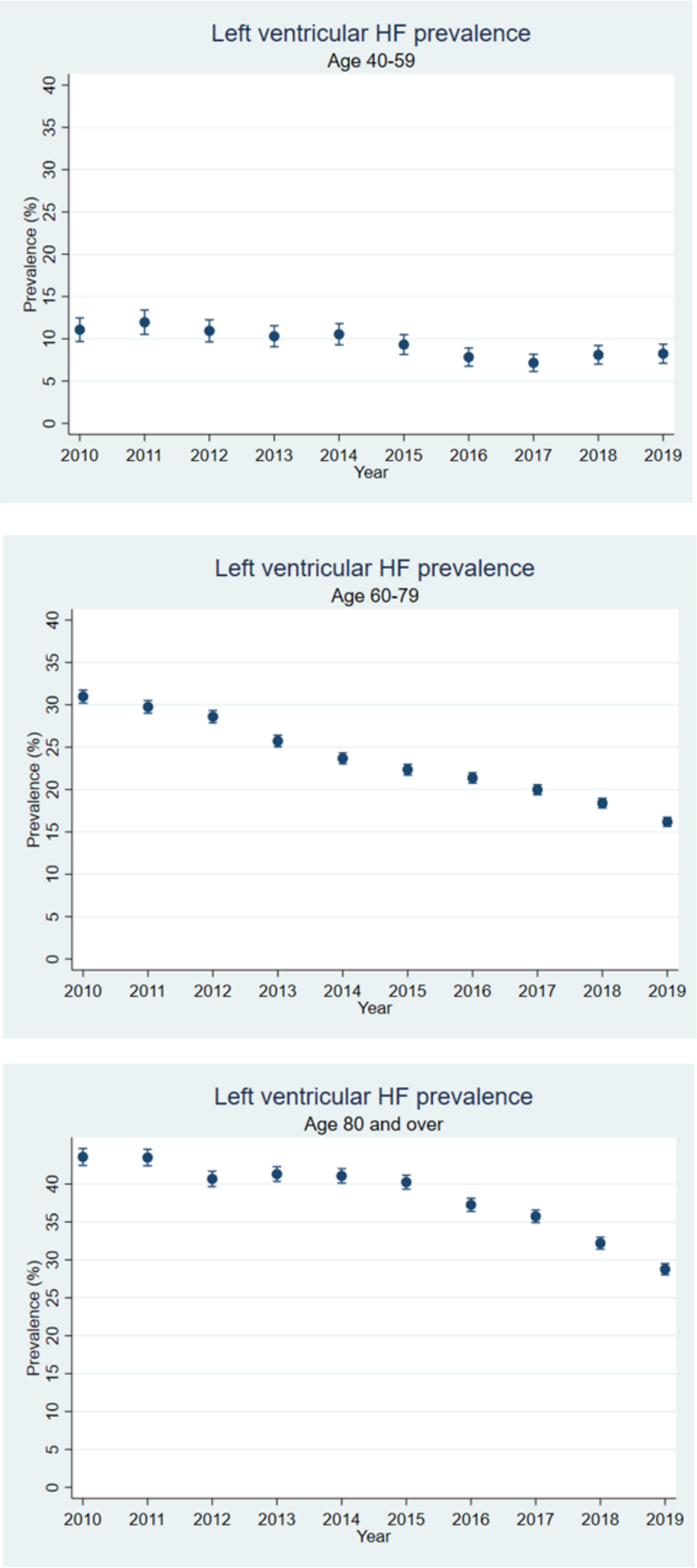

Fig S21: Annual prevalence (%) of left ventricular heart failure (HF) in patients with IPF-CS between 2010 – 2019 stratified by age. Prevalence in those aged 40-59 (top), 60-79 (middle) and 80 and over (bottom). Vertical bars for each prevalence estimate represent 95% confidence intervals.

Fig S22: Sensitivity Analyses - Left HF incidence

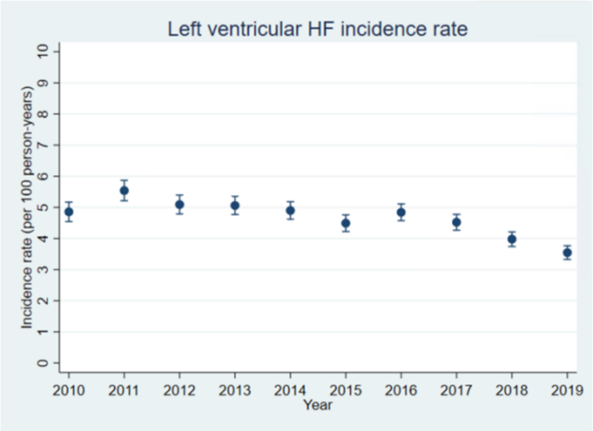

Main analysis

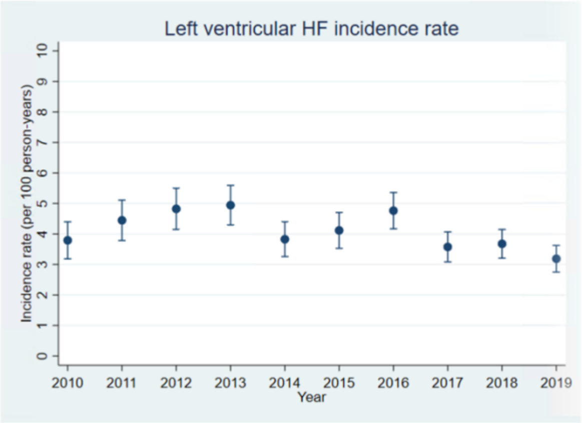

Exclusion of those with non-specific fibrosis codes

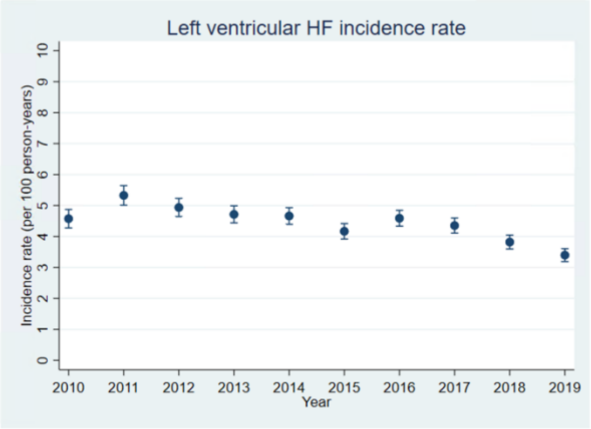

Exclusion of those with potential other causes for pulmonary fibrosis

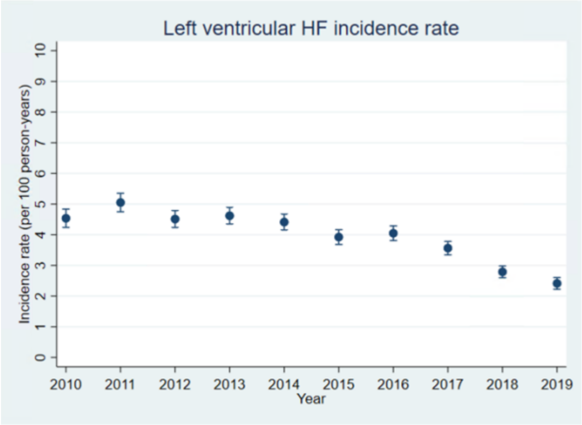

Exclusion of those diagnosed with IPF post 2018

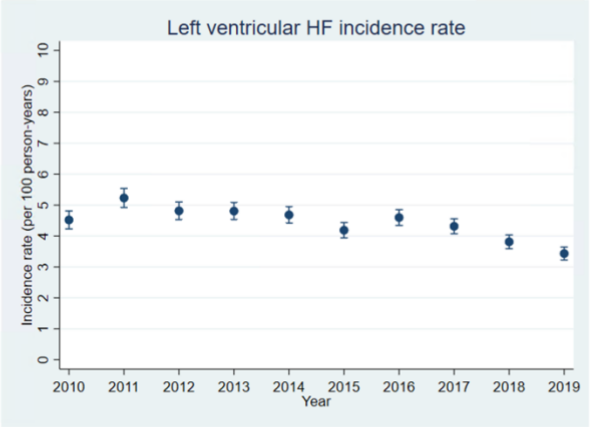

Exclusion of those diagnosed younger than age 50

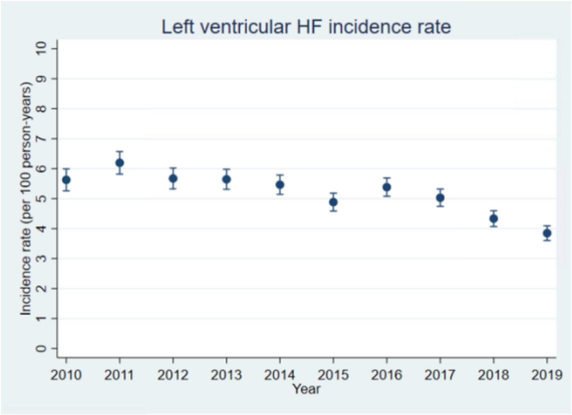

Exclusion of those diagnosed younger than age 60

Table S23: Incidence rate of Left HF per 100 person-years (95%CI) for each year of the study period when excluding those with non-specific IPF-CS codes

Year	Number of new HF cases	Person years	Incidence rate per 100 person-years (95% CI)
2010	39	1028.60	3.79 (2.70 – 5.18)
2011	45	1011.83	4.45 (3.24 – 5.95)
2012	51	1057.59	4.82 (3.59 – 6.34)
2013	58	1173.23	4.94 (3.75 – 6.39)
2014	45	1175.71	3.83 (2.79 – 5.12)
2015	49	1190.22	4.12 (3.05 – 5.44)
2016	64	1343.39	4.76 (3.67 – 6.08)
2017	53	1482.09	3.58 (2.68 – 4.68)
2018	61	1659.32	3.68 (2.81 – 4.72)
2019	53	1663.20	3.19 (2.39 – 4.17)

Table S24: Incidence rate of Left HF per 100 person-years (95%CI) for each year of the study period when excluding those with potential other causes for pulmonary fibrosis

Year	Number of new HF cases	Person years	Incidence rate per 100 person-years (95% CI)
2010	235	5137.24	4.57 (4.01 – 5.20)
2011	285	5350.12	5.33 (4.73 – 5.98)
2012	284	5752.64	4.94 (4.38 – 5.55)
2013	293	6215.13	4.71 (4.19 – 5.29)
2014	302	6477.58	4.66 (4.15 – 5.22)
2015	277	6647.82	4.17 (3.69 – 4.69)
2016	320	6972.71	4.59 (4.10 – 5.12)
2017	319	7328.89	4.35 (3.89 – 4.86)
2018	291	7617.33	3.82 (3.39 – 4.29)
2019	267	7858.40	3.40 (3.00 – 3.83)

Table S25: Incidence rate of Left HF per 100 person-years (95%CI) for each year of the study period when excluding those diagnosed with IPF-CS post change of diagnostic guidelines in 2018

Year	Number of new HF cases	Person years	Incidence rate per 100 person-years (95% CI)
2010	231	5090.13	4.54 (3.97 – 5.16)
2011	280	5544.25	5.05 (4.48 – 5.68)
2012	268	5939.52	4.51 (3.99 – 5.09)
2013	295	6385.72	4.62 (4.11 – 5.18)
2014	293	6640.47	4.41 (3.92 – 4.95)
2015	267	6804.72	3.92 (3.47 – 4.42)
2016	288	7111.89	4.05 (3.60 – 4.55)
2017	265	7437.65	3.56 (3.15 – 4.02)

2018	212	7598.91	2.79 (2.43 – 3.19)
2019	161	6671.00	2.41 (2.06 – 2.82)

Table S26: Incidence rate of Left HF per 100 person-years (95%CI) for each year of the study period when excluding those diagnosed with IPF-CS younger than 50 years old

Year	Number of new HF cases	Person years	Incidence rate per 100 person-years (95% CI)
2010	241	4962.46	4.86 (4.26 – 5.51)
2011	286	5162.27	5.54 (4.92 – 6.22)
2012	282	5538.77	5.09 (4.51 – 5.72)
2013	302	5966.69	5.06 (4.51 – 5.67)
2014	304	6204.26	4.90 (4.36 – 5.48)
2015	281	6256.07	4.49 (3.98 – 5.05)
2016	323	6671.44	4.84 (4.33 – 5.40)
2017	316	6989.09	4.52 (4.04 – 5.05)
2018	288	7240.41	3.98 (3.53 – 4.46)
2019	264	7442.97	3.55 (3.13 – 4.00)

Table S27: Incidence rate of Left HF per 100 person-years (95%CI) for each year of the study period when excluding those diagnosed with IPF-CS younger than 60 years old

Year	Number of new HF cases	Person years	Incidence rate per 100 person-years (95% CI)
2010	234	4159.00	5.63 (4.93 – 6.40)
2011	269	4342.08	6.20 (5.48 – 6.98)
2012	265	4671.61	5.67 (5.01 – 6.40)
2013	286	5066.78	5.64 (5.01 – 6.34)
2014	290	5306.66	5.46 (4.85 – 6.13)
2015	267	5467.52	4.88 (4.32 – 5.51)
2016	309	5739.86	5.38 (4.80 – 6.02)
2017	303	6023.09	5.03 (4.48 – 5.63)
2018	271	6254.65	4.33 (3.83 – 4.88)
2019	248	6444.96	3.85 (3.38 – 4.36)

Fig S28: Sensitivity Analyses - Left HF prevalence

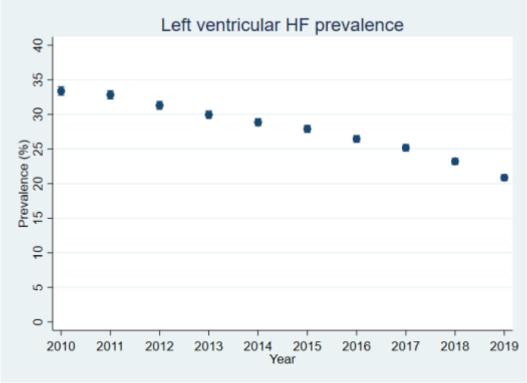

Main analysis

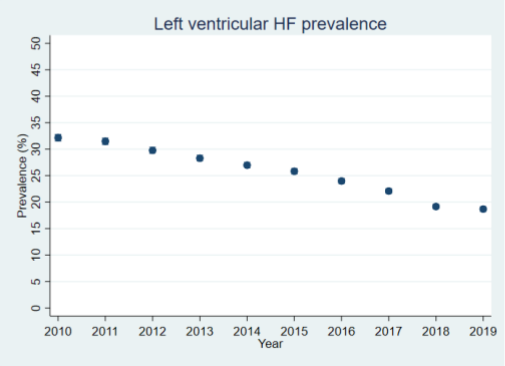

Exclusion of those diagnosed with IPF post 2018

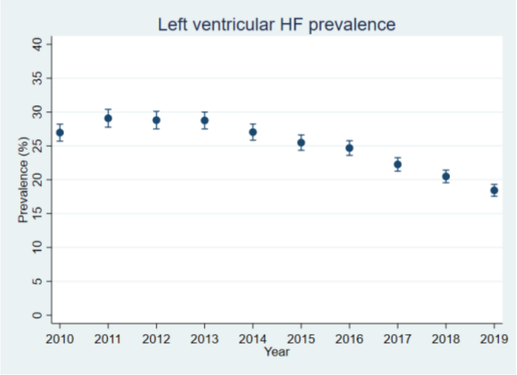

Exclusion of those with non-specific fibrosis codes

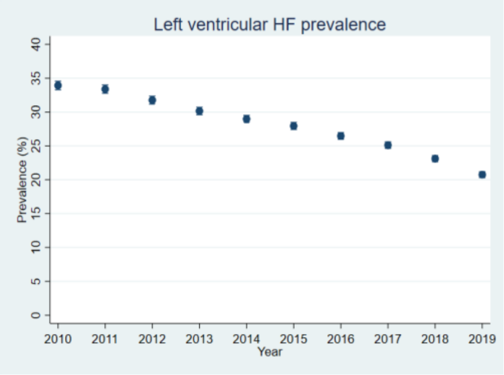

Exclusion of those with potential other causes for pulmonary fibrosis

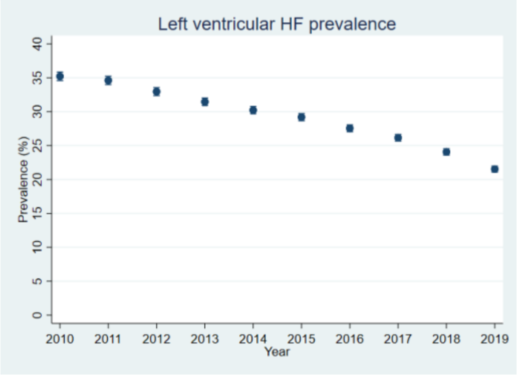

Exclusion of those diagnosed younger than age 50

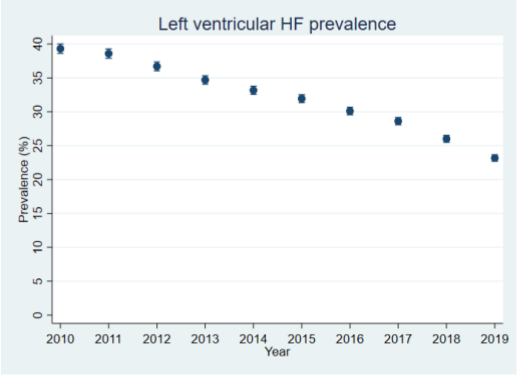

Exclusion of those diagnosed younger than age 60

Table S29: Prevalence of Left HF (%) (95%CI) in those with IPF-CS for each year of the study period when excluding those with non-specific IPF-CS codes

Year	Number of prevalent left HF cases	Total number of IPF patients	Prevalence (%) with 95% CI
2010	340	1261	27.0 (24.5 – 29.5)
2011	344	1183	29.1 (26.5 – 31.8)
2012	354	1229	28.8 (26.3 – 31.4)
2013	379	1318	28.8 (26.3 – 31.3)
2014	372	1376	27.0 (24.7 – 29.5)
2015	369	1448	25.5 (23.3 – 27.8)
2016	388	1572	24.7 (22.6 – 26.9)
2017	386	1734	22.3 (20.3 – 24.3)
2018	383	1869	20.5 (18.7 – 22.4)
2019	355	1926	18.4 (16.7 – 20.2)

Table S30: Prevalence of Left HF (%) (95%CI) in those with IPF-CS for each year of the study period when excluding those with potential other causes for pulmonary fibrosis

Year	Number of prevalent left HF cases	Total number of IPF patients	Prevalence (%) with 95% CI
2010	1983	5845	33.9 (32.7 – 35.2)
2011	2036	6099	33.4 (32.2 – 34.6)
2012	2076	6537	31.8 (30.6 – 32.9)
2013	2129	7061	30.2 (29.1 – 31.2)
2014	2136	7372	29.0 (27.9 – 30.0)
2015	2114	7565	27.9 (26.9 – 29.0)
2016	2113	7983	26.5 (25.5 – 27.5)
2017	2110	8403	25.1 (24.2 – 26.1)
2018	2017	8725	23.1 (22.2 – 24.0)
2019	1877	9045	20.8 (19.9 – 21.6)

Table S31: Prevalence of Left HF (%) (95%CI) in those with IPF-CS for each year of the study period when excluding those diagnosed with IPF-CS post change of diagnostic guidelines in 2018

Year	Number of prevalent left HF cases	Total number of IPF patients	Prevalence (%) with 95% CI
2010	1948	6056	32.2 (31.0 – 33.4)
2011	1987	6310	31.5 (30.3 – 32.7)
2012	2008	6742	29.8 (28.7 – 30.9)
2013	2052	7250	28.3 (27.3 – 29.4)
2014	2039	7556	27.0 (26.0 – 28.0)
2015	2000	7741	25.8 (24.9 – 26.8)
2016	1955	8148	24.0 (23.1 – 24.9)
2017	1885	8533	22.1 (21.2 – 23.0)

2018	1688	8806	19.2 (18.4 – 20.0)
2019	1427	7632	18.7 (17.8 – 19.6)

Table S32: Prevalence of Left HF (%) (95%CI) in those with IPF-CS for each year of the study period when excluding those diagnosed with IPF-CS when younger than 50 years old

Year	Number of prevalent left HF cases	Total number of IPF patients	Prevalence (%) with 95% CI
2010	1999	5676	35.2 (34.0 – 36.5)
2011	2048	5917	34.6 (33.4 – 35.8)
2012	2084	6323	33.0 (31.8 – 34.1)
2013	2143	6812	31.5 (30.4 – 32.6)
2014	2146	7099	30.2 (29.2 – 31.3)
2015	2124	7275	29.2 (28.2 – 30.3)
2016	2115	7677	27.6 (26.6 – 28.6)
2017	2106	8053	26.2 (25.2 – 27.1)
2018	2005	8330	24.1 (23.2 – 25.0)
2019	1854	8609	21.5 (20.7 – 22.4)

Table S33: Prevalence of Left HF (%) (95%CI) in those with IPF-CS for each year of the study period when excluding those diagnosed with IPF-CS when younger than 60 years old

Year	Number of prevalent left HF cases	Total number of IPF patients	Prevalence (%) with 95% CI
2010	1896	4823	39.3 (37.9 – 40.7)
2011	1943	5035	38.6 (37.2 – 40.0)
2012	1980	5395	36.7 (35.4 – 38.0)
2013	2031	5854	34.7 (33.5 – 35.9)
2014	2034	6132	33.2 (32.0 – 34.4)
2015	2015	6309	31.9 (30.8 – 33.1)
2016	2011	6678	30.1 (29.0 – 31.2)
2017	2006	7009	28.6 (27.6 – 29.7)
2018	1892	7273	26.0 (25.0 – 27.0)
2019	1748	7541	23.2 (22.2 – 24.2)

Table S34: Multivariate models of the association between prevalent heart failure and risk of all-cause and cardiovascular mortality in IPF-CS

	All-cause mortality adjusted HR (95% CI)	P value	CV mortality adjusted HR (95% CI)	P value
**Prevalent HF**				
No	Ref		Ref	
Yes	1.06 (1.01 – 1.11)	0.018	1.63 (1.37 – 1.94)	<0.001
**Age, per 1 yr increment**	1.04 (1.04 – 1.05)	<0.001	1.06 (1.05 – 1.07)	<0.001
**Gender**				
Male	1.31 (1.26 – 1.37)	<0.001	1.38 (1.16 – 1.65)	<0.001
**Smoking**				
Non-smoker	Ref		Ref	
Ex-smoker	1.11 (1.04 – 1.18)	0.001	1.21 (0.93 – 1.57)	0.161
Current	1.14 (1.05 – 1.23)	0.002	1.25 (0.89 – 1.75)	0.193
**BMI**				
Normal	Ref		Ref	
Underweight	2.07 (1.89 – 2.28)	<0.001	1.36 (0.80 – 2.31)	0.261
Overweight	0.81 (0.78 – 0.85)	<0.001	1.09 (0.90 – 1.33)	0.372
Obese	0.80 (0.76 – 0.85)	<0.001	1.09 (0.87 - 1.35)	0.450
**AF**	1.08 (1.02 – 1.15)	0.006	1.25 (1.02 – 1.53)	0.029
**IHD**	1.15 (1.10 – 1.20)	<0.001	1.63 (1.38 – 1.93)	<0.001
**Hypertension**	0.98 (0.94 – 1.02)	0.278	1.22 (1.03 – 1.44)	0.021
**Valve disease**	1.11 (1.04 – 1.19)	0.002	1.11 (0.87 – 1.43)	0.387
**COPD**	1.15 (1.10 – 1.21)	<0.001	1.06 (0.88 – 1.27)	0.549
**Diabetes**	1.22 (1.17 – 1.28)	<0.001	1.55 (1.31 – 1.84)	<0.001
**Anaemia**	1.08 (1.03 – 1.14)	0.002	1.32 (1.09 - 1.59)	0.004

Table S35: Multivariate models of the association between prevalent heart failure and risk of all-cause and cardiovascular mortality in IPF-CS: inclusion of ethnicity and IMD in the model as a sensitivity analysis

	All-cause mortality adjusted HR (95% CI)	P value	CV mortality adjusted HR (95% CI)	P value
**Prevalent HF**				
No	Ref		Ref	
Yes	1.05 (1.00 - 1.11)	0.049	1.59 (1.32 – 1.91)	<0.001
**Age, per 1 yr increment**	1.05 (1.04 – 1.05)	<0.001	1.06 (1.05 – 1.07)	<0.001
**Gender**				
Male	1.31 (1.25 – 1.37)	<0.001	1.45 (1.20 – 1.74)	<0.001
**Smoking**				
Non-smoker	Ref		Ref	
Ex-smoker	1.07 (1.00 – 1.14)	0.036	1.20 (0.91 – 1.58)	0.202
Current	1.09 (1.00 – 1.18)	0.043	1.20 (0.84 - 1.71)	0.317
**BMI**				
Normal	Ref		Ref	
Underweight	2.03 (1.84 – 2.24)	<0.001	1.40 (0.80 – 2.42)	0.235
Overweight	0.82 (0.78 – 0.86)	<0.001	1.11 (0.90 – 1.36)	0.325
Obese	0.79 (0.75 – 0.84)	<0.001	1.13 (0.90 – 1.43)	0.295
**Ethnicity**				
White	Ref		Ref	
Asian	0.79 (0.71 - 0.88)	<0.001	1.05 (0.72 – 1.54)	0.797
Black	0.56 (0.44 – 0.72)	<0.001	1.33 (0.68 – 2.59)	0.408
Mixed	0.76 (0.49 – 1.18)	0.228	-	-
Other	1.20 (1.04 – 1.38)	0.010	1.54 (0.88 – 2.67)	0.127
**IMD**				
1 (least)	Ref		Ref	
2	1.0 (0.94 – 1.07)	0.986	1.02 (0.78 – 1.32)	0.906
3	1.0 (0.94 – 1.07)	0.893	0.99 (0.76 – 1.29)	0.938
4	1.10 (1.03 – 1.17)	0.006	1.17 (0.90 – 1.52)	0.251
5 (most)	1.16 (1.09 – 1.24)	<0.001	1.18 (0.90 – 1.54)	0.227
**AF**	1.08 (1.02 – 1.15)	0.011	1.25 (1.01 – 1.54)	0.037
**IHD**	1.15 (1.10 – 1.20)	<0.001	1.60 (1.34 – 1.91)	<0.001
**Hypertension**	0.98 (0.94 – 1.03)	0.418	1.23 (1.03 – 1.47)	0.022
**Valve disease**	1.13 (1.05 – 1.22)	<0.001	1.17 (0.91 – 1.52)	0.228
**COPD**	1.13 (1.07 – 1.18)	<0.001	1.05 (0.86 – 1.27)	0.639
**Diabetes**	1.24 (1.18 – 1.30)	<0.001	1.53 (1.28 – 1.83)	<0.001

**Anaemia**	1.10	(1.05 – 1.16)	<0.001	1.34 (1.11 – 1.63)	0.003

Table S36: Multivariate models of the association between prevalent heart failure and risk of IPF-specific mortality in IPF-CS

	IPF-specific mortality adjusted HR (95% CI)		P value
**Prevalent HF**			
No		Ref	
Yes	0.92	(0.86 – 0.99)	0.028
**Age, per 1 yr increment**	1.04	(1.04 -1.05)	<0.001
**Gender**			
Male	1.38	(1.30 – 1.47)	<0.001
**Smoking**			
Non-smoker		Ref	
Ex-smoker	1.04	(0.95 – 1.13)	0.385
Current	0.77	(0.69 – 0.87)	<0.001
**BMI**			
Normal		Ref	
Underweight	1.85	(1.60 – 2.13)	<0.001
Overweight	0.87	(0.81 – 0.93)	<0.001
Obese	0.89	(0.82 – 0.96)	0.003
**COPD**	0.71	(0.66 – 0.76)	<0.001

Fig S37: Graphical assessment of whether proportional hazards assumption met in multivariate Cox models in main analysis comparing risk of all-cause (top), cardiovascular (middle) and IPF-specific mortality (bottom) in those with or without prevalent HF, adjusted for various covariates.

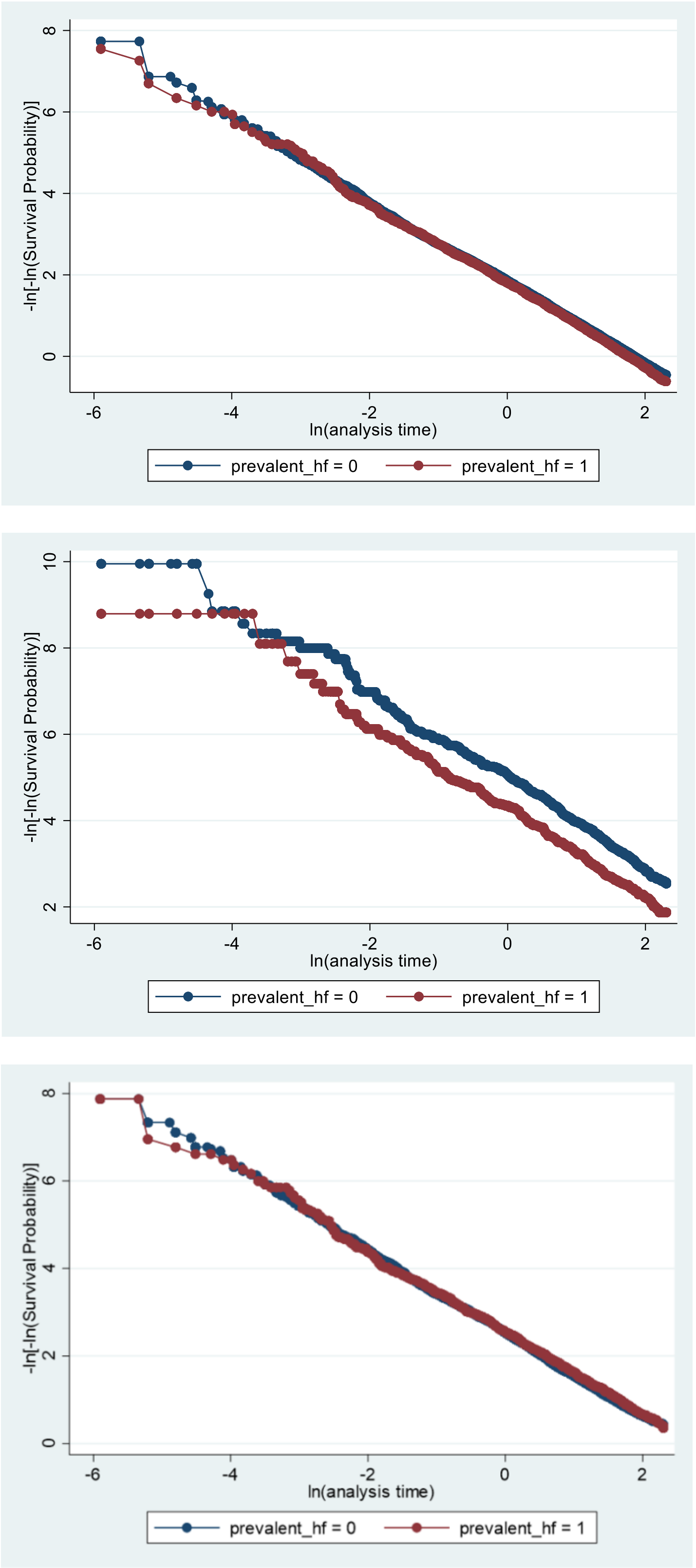

Supplement: Supplementary file 1 — Additional file 1.Supplementary material. [file 12890_2022_1973_MOESM1_ESM.pdf]
